# A systematic review and network meta-analysis on the effectiveness of exercise-based interventions for reducing the injury incidence in youth team-sport players. Part 1: an analysis by classical training components

**DOI:** 10.1080/07853890.2024.2408457

**Published:** 2024-10-01

**Authors:** Francisco Javier Robles-Palazón, Desirée Blázquez-Rincón, Alejandro López-Valenciano, Paul Comfort, José Antonio López-López, Francisco Ayala

**Affiliations:** a Department of Physical Activity and Sport, Faculty of Sport Sciences, Campus of Excellence Mare Nostrum, University of Murcia, Murcia, Spain; b Centre for Human Movement and Rehabilitation, School of Health and Society, University of Salford, Salford, United Kingdom; c Department of Psychology and Education. Faculty of Health Sciences and Education, Madrid Open University (UDIMA), Madrid, Spain; d Department of Education Science, School of Humanities and Communication Sciences, CEU-Cardenal Herrera University, Castellón de la Plana, Spain; e School of Medical and Health Sciences, Edith Cowan University, Joondalup, Australia; f Department of Basic Psychology and Methodology, Faculty of Psychology and Speech Therapy, University of Murcia, Murcia, Spain; g School of Education, Sport and Applied Sciences, University of Gloucestershire, Gloucester, United Kingdom

**Keywords:** Injury prevention, strength, flexibility, stability, adolescence, young athletes, soccer

## Abstract

**Objectives:**

The primary purposes were (a) to estimate the pooled effects of injury prevention programs (IPPs) on reducing overall and some specific body regions (lower extremity, thigh, knee, and ankle) injury incidence rates (IIRs) and (b) to compare the effects of single- and multi-component IPPs on mitigating injury risk in youth team sport athletes. A secondary objective was to explore the individual effects of different components on these IIRs.

**Materials and methods:**

Searches were performed up to 15 January 2024 in PubMed, Web of Science, SPORTDiscus, and Cochrane Library. Eligible criteria were: exercise-based interventions evaluated against a control group, overall IIRs were reported, and youth (≤19 years old) team sport players. Two reviewers extracted data and assessed trial quality using the Consolidated Standards of Reporting Trials (CONSORT) statement, the Physiotherapy Evidence Database Scale (PEDro), and a risk of bias tool (Cochrane Back and Neck Group). Pooled effects were calculated by Frequentist random effects pairwise and network meta-analyses.

**Results:**

Twenty-one studies were included. IPPs reduced overall, lower extremities, thigh, knee, and ankle IIRs by an average of approximately 35%. Most of the IPPs demonstrated statistically significant risk mitigation effects for overall and lower extremity injuries compared to control group. Interventions comprised exclusively of strength ([IRR = 0.3 [95%CI = 0.10–0.93]) and flexibility (IRR = 0.49 [95%CI = 0.36–0.68]), as well as those including stability exercises, were the most effective measures for reducing injuries in youth team sports.

**Conclusions:**

The implementation of current IPPs in training sessions for several weeks has shown to be an effective strategy for reducing the risk of injury in youth team sport athletes by one-third. Indirect evidence suggests that strength, flexibility, and stability might be exercise components with the highest risk mitigation effects; however, more research is crucial to confirm our estimates with direct evidence.

## KEY MESSAGES

- Injury prevention programs could reduce around one-third the incidence of injuries occurring in youth team sport athletes.
- Strength, flexibility, and stability seem to be key exercise-based components to be included in any training program aimed at minimizing the risk of injury in youth.
- Further research comparing head-to-head injury prevention programs in youth athletes is needed to make more founded conclusions on their true risk mitigation effects.

## 1. Introduction

Regular participation in organized sports during childhood and adolescence has demonstrated numerous health-related benefits [1,2]. However, the progressive increase in physical demands of sport competition and specialization from a young age observed in recent years has led to, among other problems, a heightened injury risk in this population [3]. This increased injury risk of youth athletes has been documented in a variety of age ranges, levels of performance, and team sports (including soccer [4], rugby [5], and handball [6]). For example, a recent meta-analysis on the epidemiology of injuries in youth soccer has reported an injury probability during a competitive season of 47% and 43% for male and female players, respectively [4]. Several sport-related injuries (e.g. ankle sprains and thigh muscle strains) have often an adverse and temporary impact on youth athletes' health and well-being [7]. Furthermore, injuries may also present negative effects on long-term youth athlete development due to recurrent and/or prolonged absence from sports participation [8], and a few of them (e.g. ruptures of the anterior cruciate ligament [ACL] of the knee) may even predispose youth athletes to early sport termination or dropout and/or compromise function in later life, limiting the ability to experience pain-free mobility and engage in health-enhancing activities [9]. Finally, sport-related injuries may represent a significant financial burden in terms of treatment (possible surgery for some injuries) and rehabilitation (i.e. physical therapy) costs [10]. Therefore, these figures emphasize the urgent need for delivering effective risk mitigation strategies to youth team sport athletes.

Multiple exercise-based strategies, typically short duration (i.e. 10–30 min) training programs (also known as injury prevention programs [IPPs]), have been developed (in the form of standardized warm-up protocols [e.g. FIFA 11 + [11], FIFA 11+ Kids [12]] or independent interventions [e.g. balance training [13,14]) to be delivered in training sessions for several weeks to reduce the number of injuries sustained by youth athletes. The exercises integrated into these IPPs usually target evidence-based modifiable risk factors for lower extremity injuries (the anatomical region where injuries most frequently occur in youth team sports) including abnormal biomechanical movement patterns (e.g. excessive dynamic knee valgus motion at landing and cutting) and neuromuscular deficits (e.g. muscle weakness and poor dynamic joints stability) [11–14]. The results of several systematic reviews and meta-analyses have consistently demonstrated the effectiveness of IPPs not only in reducing injury incidence but also in increasing physical performance when adequate intervention compliance is achieved in youth team sport athletes [15,16]. However, it should be highlighted that these systematic reviews and meta-analyses have also shown that the magnitude of injury risk mitigation effects varies widely among IPPs, from no significant effect up to 70% reduction in injury rates [17,18]. IPPs are usually comprised of one [19,20] or multiple [11,12] exercise components (e.g. strength, plyometrics, change of direction, stability, flexibility). Therefore, it might be suggested that the IPP modality (single exercise component [S-IPPs] *vs*. multiple exercise components [M-IPPs]) but mainly the type/s of exercise component of IPPs (e.g. strength, stability) are features that might partially explain this heterogeneity observed among primary studies regarding up to what extent IPPs are effective exercise-based strategies to reduce the risk of injury. These primary studies have mainly conducted direct (i.e. pair-wised) comparisons between the injury incidence rates reported by an intervention group that carried out always the same IPP (e.g. a M-IPP comprised of stability and flexibility exercises) and a control group that performed standard practices or sham exercises [11–14,19,20]. Due to the lack of primary studies that had examined the individual injury risk mitigation effects of two or more IPPs, the use of statistical techniques that simultaneously allow making direct and indirect comparisons among multiple interventions (IPPs) considering the 'full network' of available trials is required to address the question regarding what type/s of exercise component of IPPs yield the greatest reduction in injury risk.

Some systematic reviews and meta-analyses have been recently published exploring the most effective combinations of exercise components for IPPs to prevent injuries in different cohorts of team-sport athletes [21,22]. However, the use of classical meta-analytical techniques may make it difficult to draw an overall conclusion on this issue because, among other limitations, they only have the ability to compare no more than two groups or conditions (e.g. intervention group [S-IPP comprised by flexibility exercises] *vs*. control group) at a time using data from primary studies in which direct comparisons between these two had been conducted [23]. Furthermore, it is difficult to adjust for differences in participants (e.g. athletes engaged in different team sports) and study-level characteristics among included trials when analyses are conducted in a separate fashion, and it is also limiting in terms of assessing whether (or not) findings are internally consistent [23].

Network meta-analysis is an advanced methodology for research synthesis that allows pooling evidence on multiple interventions (e.g. IPPs) from a set or network of primary studies that include a common comparator (i.e. control group) through a mixed comparison approach (i.e. a weighted average of the direct and indirect estimates of intervention effects) [24,25]. Network meta-analysis provides a more inclusive approach than classical meta-analysis since all pairwise comparisons of interventions can be simultaneously examined within a single analysis enabling the estimation of their relative ranking and hierarchy for a given outcome (e.g. sport-related injury incidence in youth team sport athletes) [26]. Component-level network meta-analysis regression methods have been recently developed to allow estimation of the additive contribution of components and/or combinations of components of complex interventions such as IPPs [27]. Although this novel approach has the potential to address the research question stated before and optimize future injury prevention practices, no network meta-analyses have been conducted (to the best of the authors' knowledge) in this field.

The primary purposes of this systematic review and network meta-analysis were therefore (a) to estimate the pooled effects of IPPs on reducing overall and some specific body regions (lower extremity, thigh, knee, and ankle) injury incidences, and (b) to compare the effects of S-IPPs and M-IPPs on mitigating injury risk in youth team sport athletes. A secondary objective was to explore the individual effects of different exercise-based components (i.e. strength, plyometrics, stability, speed and agility, coordination/warm-up drills, and flexibility) on the injury incidences previously mentioned.

## 2. Methods

This systematic review and component-level network meta-analysis were carried out following the Preferred Reporting Items for Systematic Reviews and Meta-Analysis (PRISMA) guidelines [28] along with the specific extension for network meta-analyses (PRISMA-NMA) [29]. The PRISMA-NMA checklist is presented in online supplementary file 1. The research protocol was registered with the PROSPERO International Prospective Register of Systematic Reviews (http://www.crd.york.ac.uk/PROSPERO/), registration number CRD42020152487.

### 2.1. Study selection

Eligibility criteria were established and agreed upon by all authors based on the concept of population, intervention/indicator, comparator/control, and outcome (PICOS) [29] (for more information, please see online supplementary file 2). Thus, to be included in this systematic review and meta-analysis, studies were required to be full-text articles published in a peer-reviewed journal before January 2024 and satisfy the following criteria:

- The study population consisted of youths (males and females) of 19 years or younger, participating in structured/organized team sports programs on a competitive level (P).
- An IPP (defined as exercise-based strategies comprised of one [S-IPP] or multiple exercise component [M-IPP] IPP [both warm-ups' protocols and independent interventions] that had the aim of reducing injury incidence) was evaluated with no co-interventions (e.g. education) provided (I).
- The study contained a control group of similar-age participants either performing usual practice routine or sham exercises without a specific focus on modifiable lower extremity injury risk factors (e.g. neuromuscular control) but still was exposed to normative existing practices (C).
- Epidemiological data (injury incidence, number of injuries, and/or hours of sport exposure) of overall injuries (i.e. the total number of injuries prospectively recorded through the follow-up period of the study) were provided. Injuries were considered in accordance with Fuller et al.'s [30] time-loss and medical attention definitions. Thus, all types of injuries that fitted these definitions were included (O).
- An analytical prospective design was used (RCTs, quasi-experimental trials, cohort, and observational studies) (S).

When eligibility could not be confirmed from the reported data, the authors were contacted for additional information. Interventions using protective devices (i.e. braces, tapes), literature reviews, abstracts, editorial commentaries, and letters to the editor were excluded. Articles not peer-reviewed or not written in English or Spanish were also excluded. Finally, studies reporting incidences for specific injuries (e.g. anterior cruciate ligament of the knee tears, hamstring muscle strains), but not for overall and/or lower extremity incidents, were discarded.

Given that the data in this study were collected from previous trials where participants had already provided informed consent, ethical approval from a research ethics committee was not required for this investigation.

### 2.2. Search strategy

A systematic computerized search was conducted up to 15 January 2024 in the databases PubMed, Web of Science, SPORTDiscus, and Cochrane Library. In addition, a complementary search of the reference lists of included articles and a Google Scholar search were also performed. This was done using backward citation tracking (to manually search the reference list of a journal article), and forward citation tracking (scanning a list of articles that had cited a given paper since it was published) [31]. Citations were tracked using Google Scholar to make sure that studies were not missed inadvertently. When additional studies that met the inclusion criteria were identified, they were included in the final pool of studies. Relevant search terms were used to construct Boolean search strategies, which can be found in the online supplementary file 3. No limitations were imposed on the date of publication.

Two reviewers independently (FJR-P and AL-V) selected studies for inclusion in a two-step process. First, studies were screened based on title and abstract. In the second stage, full-text studies were reviewed to identify those studies that met the eligibility criteria. A study was excluded immediately once it failed to meet a single inclusion criterion. Disagreements were resolved through consensus or by consulting a third reviewer (FA).

### 2.3. Data extraction

A codebook was produced to standardize the coding of each study to maximize objectivity, and each study was coded by two different reviewers. The moderator variables of the eligible studies were coded and grouped into four categories: 1) general study descriptors (e.g. authors, year of publication, and study design), 2) study population (e.g. sample size, sex, team sport, and level or standard of sport participation), 3) characteristics of the interventions (e.g. length, duration, IPP modality, equipment required, who delivered the intervention, and type/s of exercise component/s integrated into the IPP) and 4) epidemiological data (e.g. number of injuries, exposure hours [training and match], and injury incidence). If applicable, the authors of included studies were contacted to provide clarifications or access to raw data. Online supplementary file 4 displays the moderator variables coded separately by category.

For the primary purpose of this network meta-analysis, the incidence was extracted for reported 'overall or total injuries'. If the incidence was not reported, it was calculated by dividing the number of injuries by the total hours of exposure for each intervention and control groups. The number of injuries by anatomic location, type of injury, severity, and mechanisms according to the operational definitions reported by Fuller et al. [30] were also recorded to explore possible sub-analyses.

Regarding the category type/s of exercise component/s integrated into the IPP, seventeen different labels were defined: 1) control (standard practices), 2) strength, 3) stability, 4) plyometrics, 5) flexibility, 6) plyometrics + stability, 7) strength + plyometrics + stability + speed and agility + warm-up drills, 8) strength + plyometrics + stability, 9) strength + plyometrics + stability + speed and agility, 10) strength + plyometrics + stability + speed & agility + warm-up drills + flexibility, 11) stability + flexibility, 12) strength + stability,13) strength + plyometrics + stability + warm-up drills + flexibility, 14) strength + plyometrics, 15) stability + warm-up drills + flexibility, 16) plyometrics + speed and agility + warm-up drills, and 17) plyometrics + stability + speed and agility + warm-up drills + flexibility. Labels 2–5 are single exercise components that were adapted from previous studies [22,32,33]. A detailed description of each single exercise component is provided in the online supplementary file 4. In particular, the conceptual definitions of labels 2–4 (including examples of activities belonging to them) were founded on the taxonomy of integrative neuromuscular training components described by Fort-Vanmeerhaeghe et al. [32]. Each study was classified as including an exercise component if they described at least one activity pertaining to the component definition. Furthermore, individual components (strength, stability, plyometrics, flexibility, speed and agility, and warm-up drills) were assessed against their respective training prescription recommendations [34–39] to determine if they met the required criteria to provide an effective stimulus. If studies had unclear reporting of the intervention activities, referenced work of the intervention was examined where available. Labels 6–17 are different combinations of exercise components in the same M-IPP. It should be noted that the order of appearance of every single component in M-IPPs was not considered but just its presence. These combinations of exercise components were chosen as they are the most frequently observed in M-IPPs (e.g. FIFA 11+ = strength + plyometrics + stability + speed and agility + warm-up drills).

### 2.4. Quality and risk of bias assessments

The reporting quality of included studies was assessed using the 'Consolidated Standards of Reporting Trials' (CONSORT) statement by Schulz et al. [40]. Online supplementary file 5 displays a description of the 25 criteria designed to assess the quality of the studies included in the meta-analysis with the CONSORT scale. Although the CONSORT statement was not developed to directly assess the quality of publications, compliance with the CONSORT checklist has been recognized as a proxy for the quality of the publications on randomized control trials since there is no validated instrument for this purpose [41]. The items and subitems of the CONSORT statement were scored as 0 or 1, with a score of 1 provided for each checklist item that was properly completed. Using this checklist, a maximum score of 35 would indicate the article fulfilled the requirements for a high-quality publication.

The methodological quality of the studies selected was evaluated using the Physiotherapy Evidence Database Scale (PEDro) [42] (online supplementary file 6). The PEDro scale has been demonstrated to be reliable in clinical and randomized trials [43] and has been used in several intervention meta-analyses [18,33,44]. Each study's total score out of 10 is derived, adding the satisfied criteria. A PEDro score ranging from 6 to 10 is indicative of high quality, whereas scores of 4–5 indicate fair quality, and scores of 3 or less indicate poor quality [44].

Furthermore, to assess the risk of bias of external validity quality, the scale for experimental studies with a control group designed by Cochrane Back and Neck Group was used [45]. The types of biases assessed were selection bias (criteria 1, 2, 9), performance bias (criteria 3, 4, 10, 11), attrition bias (criteria 6, 7), detection (or measurement) bias (criteria 5, 12) and reporting bias (criterion 8) (online supplementary file 7). The last criterion, 'Other' (criteria 13), was reserved for any type of potential bias that was not detected by the previous items. The higher the number of 'yes' given to an article, the lower the risk of bias. In the scientific literature, several tools have been described to assess the risk of bias in intervention studies' results. The most popular tools are RoB 2 for randomized trials [46] and ROBINS-I for non-randomized trials [47]. However, these tools provide an overall qualitative judgment of the risk of bias in the results of intervention studies using a taxonomy that comprises three (low, high, and some concerns) and five (low, moderate, serious, critical, and no information) categories for RoB 2 and ROBINS-I tools, respectively, depending on the qualitative scores obtained in each tool's included domains. Both tools have high requirements to consider an intervention study as having a low risk of bias in its results. For instance, a study is considered at low risk of bias only if all domains in RoB 2 are judged as low risk of bias. Furthermore, some domains in these tools, such as domains 2 (risk of bias due to deviations from the intended interventions) and 4 (risk of bias in the measurement of the outcome) of RoB, are difficult to satisfy in the context of IPPs. These two aspects, along with the results reported in the systematic review conducted by Fanchini et al. [48] on the effects of IPPs on muscle injuries in soccer, led the authors to conclude that most or even all of the eligible studies for the current systematic review and meta-analysis would be judged as having a high (randomized trials) and critical (non-randomized trials) risk of bias in their results. Therefore, conducting a moderator analysis using the risk-of-bias judgments of the selected studies as the dependent variable was not possible. In fact, the variable formed by these scores could not be considered a dependent variable but a constant. However, these two tools would be extremely valuable to carry out a detailed analysis of the risk of bias in the results of IPP studies through a systematic review using a best evidence synthesis approach, which is beyond the scope of the current study. Such an analysis could be done by examining each domain separately and synthesizing the evidence to draw conclusions about the overall risk of bias in the results of IPP studies.

The data extraction and quality assessments (including the risk of bias) were conducted by two reviewers (FJR-P and AL-V). To assess the inter-coder reliability of the coding process, these two reviewers (FJR-P and AL-V) coded 11 studies randomly (52%) (including quality assessment). For the quantitative moderator variables, intra-class correlation coefficients (ICC_3,1_) were calculated, while Cohen's kappa coefficients were applied for the qualitative moderator variables. On average, the ICC was 0.88 (range: 0.77 − 1.0), and the kappa coefficient was 0.81 (range: 0.63 − 1.0), which can be considered highly satisfactory, as proposed by Orwin and Vevea [49]. Inconsistencies between the two coders were resolved by consensus, and when these were due to ambiguity in the coding book, this was corrected. As before, any disagreement was resolved by mutual consent in consultation with a third reviewer (FA).

### 2.5. Statistical analyses

The statistical analysis was structured into three different stages or levels of concretion, moving from the most general to the most specific aspects, addressing in each of them one of the purposes of this study. Thus, in the first level of concretion, the estimation of pooled effects of IPPs on reducing overall and some specific body region injury incidences (including all S-IPPs and M-IPPs together in the same analysis) was conducted through random effects classical meta-analyses separately for each outcome and comparison. Heterogeneity was assessed through prediction intervals, alongside the examination of the *Q* statistic and the *I*^2^ index. Afterward, in the second level of concretion, the different programs were compared by fitting a random-effects network meta-analysis model for each outcome. Finally, in the third stage, a random-effects network meta-analysis model at a component level was run for each type of injury to explore the individual effects of the different exercise-based components defined (i.e. strength, plyometrics, stability, speed & agility, coordination/warm-up drills, and flexibility) on each outcome.

Two different approaches were used for data analysis in the second and third levels of concretion:

1. Comprehensive analysis: considered a single exercise component (e.g. strength) to be present in an IPP if it included at least one exercise (e.g. body-weight squat) used to improve that physical component, regardless of training load (i.e. sets, repetitions, intensity, and/or duration). This analysis attempts to determine the effect of the mere presence of the components in the intervention.
2. Restrictive analysis: considered a single exercise component (e.g. flexibility) to be included in an IPP only if its training load (1 set of 30 seconds or 15 repetitions of static and dynamic stretching, respectively) met minimum exercise prescription guidelines for producing desirable effects (e.g. increases in hip flexion range of motion) in youth athletes. This analysis aims to determine the effect of including the recommended dose for that component in the intervention.

The restrictive approach led to a modification in the consideration of exercise-based components that comprised the M-IPPs in the selected articles. Specifically, some M-IPPs lost one or two exercise-based components because they did not meet the minimum training recommendations outlined previously [34–39].

#### 2.5.1. Pooled effects on reducing overall and some specific body region injury incidences

Injury incidence rates (IIRs) per 1000 h of player exposure were extracted from the included studies. If IIRs were not specifically reported, they were, if possible, calculated from the available raw data using the following formula: IIR = 1000 × (∑injuries/∑exposure hours).

Similar to previous meta-analyses on the effectiveness of IPPs in reducing sport-related injuries [18,22,33], data were modeled using a classical, traditional meta-analysis in this first level of concretion. The response variable in each classical meta-analysis (overall, lower extremity, thigh, knee, and ankle injuries) conducted was the pooled estimated effect size of the IPPs expressed through the injury incidence rate ratio (IRR). For IRR, the overall estimated means for each random effect factor were obtained from the model and then back-transformed to give the IRR, along with 95% confidence intervals.

The heterogeneity exhibited by the pooled IRR, which represents the percentage of total variation across all studies due to between-study heterogeneity, was assessed by constructing a forest plot and by calculating the *Q* statistic and the *I*^2^ index.

Analyses of potential moderator variables were carried out when there were at least 10 estimates. Thus, the possible influence of the following qualitative/categorical and quantitative/numeric potential moderators on the models was analyzed independently through analyses of variance (ANOVAs) and simple meta-regressions respectively assuming a random-effects model in both cases: study design (randomized *vs*. non-randomized), equipment (i.e. material resources such as elastic bands, strength bars, and dumbbells) needed to carry out the IPP (yes *vs*. no), who monitor the execution of the IPP through the intervention phase (researcher/s *vs*. trainer *vs*. players themselves), male rate, length (weeks) of the IPP, duration (min) of each IPP, CONSORT score, PEDro score, and risk of bias score.

Finally, to assess the generalizability of our results, an inspection of the funnel plot asymmetry and Egger's regression test were applied to detect the potential threat of publication bias. Both methods were applied to the classical meta-analyses performed for the five types of injury analyzed in this study.

Both pair-wise and network meta-analyses were carried out within a Frequentist framework. All resulting networks were star-shaped, so that there was no potential for inconsistency among direct and indirect evidence. The analyses were performed in *R*, using the metafor and netmeta packages [50–52]. The analysis codes are available in https://data.mendeley.com/datasets/njzj278dz3/2.

## 3. Results

### 3.1. Descriptive characteristics of the studies

**Figure 1.**
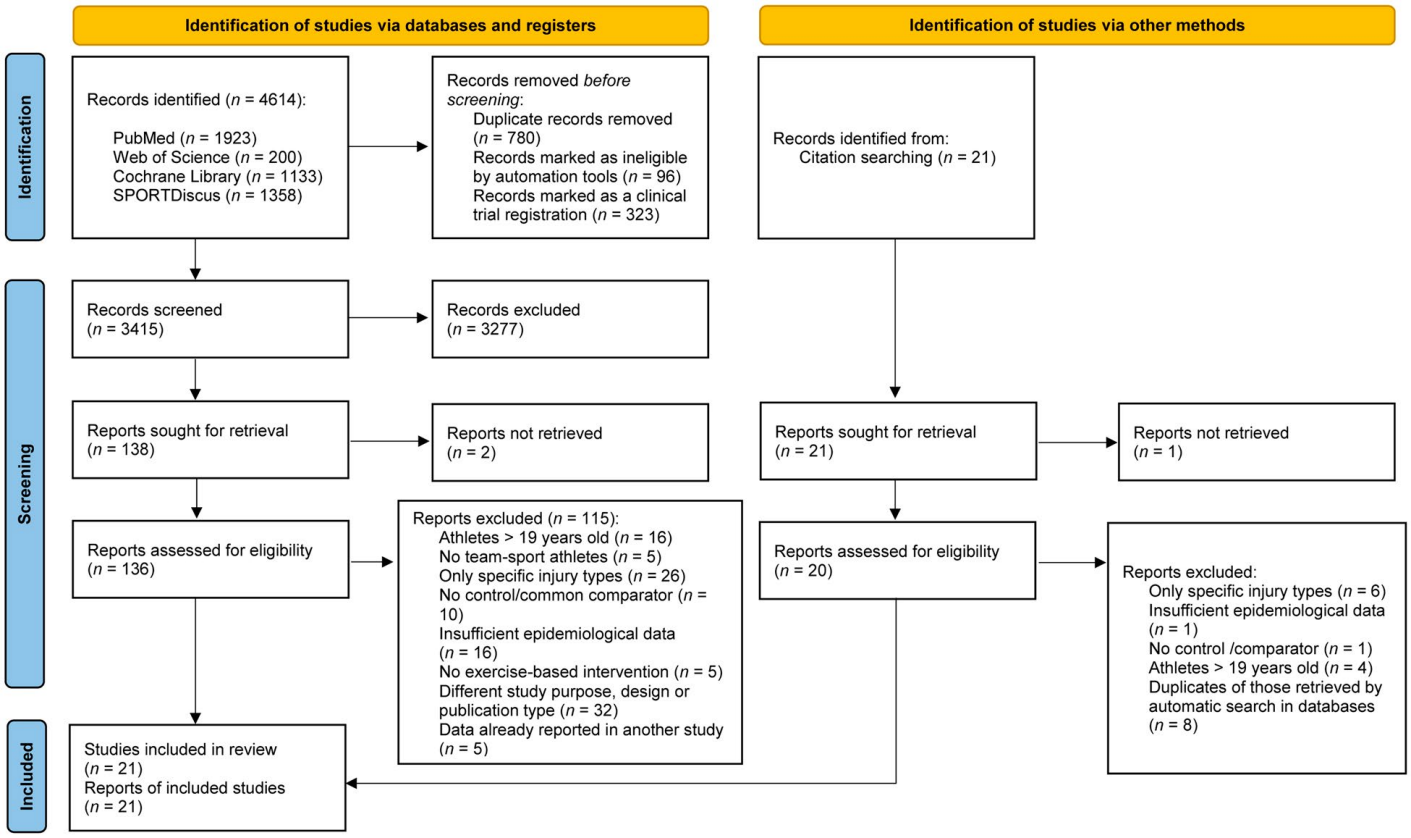
PRISMA flow diagram of the selection of studies for this systematic review and network meta-analysis.

A total of 4614 references were identified with all search strategies, of which 21 met the inclusion criteria [11–14,19,20,53–67]. Figure 1 shows the flow chart of the selection process of the studies.

Table 1 provides a descriptive summary of the main characteristics of the 21 studies finally included in this systematic review and network meta-analysis. These studies were carried out between 1999 and 2023. The total sample size was 18,305 youth team sport athletes, 9721 for the intervention groups, and 8584 for the control groups. The sample size varies substantially among studies, from 52 (61) up to 3895 [12]. In eight studies, both male and female athletes were examined [12–14,55,63–66], while ten studies focused on male athletes [19,20,53,54,56,59–62,67], and three trials were on females only [11,57,58]. The mean age of the participants varied between 10 (62) and 17.8 years [56]. All trials studied team sport athletes, with soccer [11,12,14,19,20,53,56,57,59–62] being the most common (11,179 from the total of 18,305 participants were soccer players).

**Table 1.** Characteristics of the studies included.

References	Study design	Participants			Type of intervention	Intervention dosage	Overall IIR per 1000 h
*Country*		Sample size, *sport*	Sex	Age			
Achenbach et al. [64] *Germany*	RCT	IG: 168 players (13 teams) CG: 111 players (10 teams) *Handball*	Mixed	IG: 14.9 ± 0.9 y CG: 15.1 ± 1 y	M-IPP	L: 46 weeks F: 1.4 days / week D: 15 min	IG: 1.9 CG: 1.8
Åkerlund et al. [65] *Sweden*	RCT	IG: 301 players (31 teams) CG: 170 players (16 teams) *Floorball*	Mixed	IG: 13.6 ± 1.1 y CG: 13.2 ± 1.3 y	M-IPP	L: 26 weeks F: 1.5 days / week D: 20 min	IG: 12.1 CG: 18.7
Al Attar et al. [62] *Saudi Arabia*	RCT	IG: 377 players (45 teams) CG: 363 players (43 teams) *Soccer*	Male	IG: 7–13 (range) CG: 7–13 (range)	M-IPP	L: 24 weeks F: 2 days / week D: 17.5 min	IG: 0.9 CG: 2
Azuma and Someya [19] *Japan*	RCT	IG: 64 players (− teams) CG: 60 players (− teams) *Soccer*	Male	IG: 16.2 ± 0.8 y CG: 16.2 ± 0.8 y	S-IPP	L: 12 weeks F: 3 days / week D: 25 min	IG: 2 CG: 4
Barboza et al. [66] *Netherlands*	N-RCT	IG: 135 players (10 teams) CG: 156 players (12 teams) *Field hockey*	Mixed	IG: 11.5 ± 1.5 y CG: 12.9 ± 1.9 y	M-IPP	L: 40 weeks F: 1.4 days / week D: 12 min	IG: 4.1 CG: 6.4
Emery et al. [14] *Canada*	RCT	IG: 380 players (32 teams) CG: 364 players (28 teams) *Indoor soccer*	Mixed	IG: 13–18 (range) CG: 13–18 (range)	M-IPP	L: 20 weeks F: − D: 30 min	IG: 2.1 CG: 3.4
Emery et al. [13] *Canada*	RCT	IG: 494 players (47 teams) CG: 426 players (41 teams) *Basketball*	Mixed	IG: 13–18 (range) CG: 12–18 (range)	M-IPP	L: 18 weeks F: − D: 35 min	IG: 3.3 CG: 4
Hislop et al. [67] *United Kingdom*	RCT	IG: 1325 players (44 teams) CG: 1127 players (39 teams) *Rugby*	Male	IG: 16 ± 1.2 y CG: 15.9 ± 1.1 y	M-IPP	L: 14 weeks F: 2 days / week D: 15 min	IG: 7.8 CG: 8.1
Imai et al. [20] *Japan*	N-RCT	IG: 36 players (1 team) CG: 38 players (1 team) *Soccer*	Male	IG: 12–14 (range) CG: 12–14 (range)	S-IPP	L: 32 weeks F: 6 days / week D: 5 min	IG: 2.7 CG: 4.9
Junge et al. [53] *Switzerland*	N-RCT	IG: 101 players (7 teams) CG: 93 players (7 teams) *Soccer*	Male	IG: 16.7 ± 1.2 y CG: 16.3 ± 1.2 y	M-IPP	L: 48 weeks F: − D: −	IG: 6.7 CG: 8.5
Longo et al. [54] *Italy*	RCT	IG: 80 players (7 teams) CG: 41 players (4 teams) *Basketball*	Male	IG: 13.5 ± 2.3 y CG: 15.2 ± 4.6 y	M-IPP	L: 36 weeks F: 2 days / week D: 20 min	IG: 0.6 CG: 1.3
Olsen et al. [55] *Norway*	RCT	IG: 958 players (61 teams) CG: 879 players (59 teams) *Handball*	Mixed	IG: 16.3 ± 0.6 y CG: 16.2 ± 0.6 y	M-IPP	L: 32 weeks F: 1 day / week D: 17.5 min	IG: 1.1 CG: 2.2
Owoeye et al. [56] *Nigeria*	RCT	IG: 212 players (10 teams) CG: 204 players (10 teams) *Soccer*	Male	IG: 17.8 ± 0.9 y CG: 17.5 ± 1.1 y	M-IPP	L: 24 weeks F: 2 days / week D: 20 min	IG: 0.7 CG: 1.5
Rössler et al. [12] *Switzerland, Germany, Czech Republic and Netherlands*	RCT	IG: 2066 players (128 teams) CG: 1829 players (115 teams) *Soccer*	Mixed	IG: 10.8 ± 1.4 y CG: 10.7 ± 1.4 y	M-IPP	L: 40 weeks F: 2 days / week D: 17.5 min	IG: 1 CG: 1.6
Soligard et al. [11] *Norway*	RCT	IG: 1055 players (52 teams) CG: 837 players (41 teams) *Soccer*	Female	IG: 15.4 ± 0.7 y CG: 15.4 ± 0.7 y	M-IPP	L: 32 weeks F: 2 days / week D: 20 min	IG: 3.2 CG: 4.7
Steffen et al. [57] *Norway*	RCT	IG: 1073 players (58 teams) CG: 947 players (51 teams) *Soccer*	Female	IG: 15.4 ± 0.8 y CG: 15.4 ± 0.8 y	M-IPP	L: 25 weeks F: 1 day / week D: 20 min	IG: 3.6 CG: 3.7
Verhagen et al. [63] *Netherlands*	RCT	IG: 282 players (35 teams) CG: 236 players (31 teams) *Volleyball*	Mixed	IG: 12.9 ± 1.6 y CG: 12.6 ± 1.7 y	M-IPP	L: 28 weeks F: 2 days / week D: 15 min	IG: 1.6 CG: 2.7
Wedderkopp et al. [58] *Denmark*	RCT	IG: 111 players (11 teams) CG: 126 players (11 teams) *Handball*	Female	IG: 16–18 (range) CG: 16–18 (range)	M-IPP	L: 40 weeks F: − D: 12.5 min	IG: 1 CG: 3.7
Zarei et al. [59] *Iran*	RCT	IG: 34 players (2 teams) CG: 32 players (2 teams) *Soccer*	Male	IG: 15.3 ± 0.6 y CG: 15.5 ± 0.7 y	M-IPP	L: 30 weeks F: 3.3 days / week D: 20 min	IG: 2.9 CG: 4.3
Zarei et al. [60] *Iran*	RCT	IG: 443 players (15 teams) CG: 519 players (17 teams) *Soccer*	Male	IG: 12.1 ± 1.8 y CG: 12.2 ± 1.7 y	M-IPP	L: 36 weeks F: 2 days / week D: 20 min	IG: 0.9 CG: 1.9
Zouita et al. [61] *Tunisia*	RCT	IG: 26 players (− teams) CG: 26 players (− teams) *Soccer*	Male	IG: 13–14 (range) CG: 13–14 (range)	S-IPP	L: 12 weeks F: 2.5 days / week D: 90 min	IG: 0.8 CG: 2.7

L: length; F: frequency, D: duration; S-IPP: Single-component injury prevention program; M-IPP: Multicomponent injury prevention program; RCT: Randomized controlled trial; N-RCT: Non-randomized controlled trial; IG: Intervention group; CG: Control group; IIR: Injury incidence rate.

### 3.2. Type and components of the injury prevention programs

Supplementary file 8 summarizes the characteristics of the IPPs included in the overall analysis. The specific exercises included in each study were highly wide-ranging. M-IPP was the most common intervention used in 18/21 studies [11–14,53–60,62–67]; conversely, three studies analyzed S-IPPs [19,20,61]. The most common exercise components in IPPs were stability (19/21 studies) [11–14,20,53–60,62–67] and strength (18/21 studies) [11,12,14,53–67] while flexibility exercises were used the least (6/21 studies) [13,14,19,53,63,66]. Concerning meeting training prescription guidelines, 61% (11/18), 81% (13/16), 68% (13/19), 70% (7/10), 89% (8/9), and 67% (4/6) met the guidelines for strength, plyometrics, stability, speed and agility, warm-up drills, and flexibility, respectively (Table 2).

**Table 2.** Intervention components according to training prescription guidelines.

References	Strength	Plyometrics	Stability	Speed/agility	Coordination / warm-up drills	Flexibility
Achenbach et al. [64]	✓	✓	✓	x	x	x
Åkerlund et al. [65]	✓	✓	✓	x	x	x
Al Attar et al. [62]	✓	✓	✓	x	x	x
Azuma and Someya [19]	x	x	x	x	x	✓
Barboza et al. [66]	✓	✓	✓	✓	✓	✓
Emery et al. [14]	✓	✓	✓	x	✓	✓
Emery et al. [13]	x	x	✓	x	x	✓
Hislop et al. [67]	✓	✓	✓	✓	x	x
Imai et al. [20]	x	x	✓	x	x	x
Junge et al. [53]	✓	✓	✓	✓	✓	✓
Longo et al. [65]	✓	✓	✓	✓	✓	x
Olsen et al. [55]	✓	✓	✓	✓	✓	x
Owoeye et al. [56]	✓	✓	✓	✓	✓	x
Rössler et al. [12]	✓	✓	✓	x	x	x
Soligard et al. [11]	✓	✓	✓	✓	✓	x
Steffen et al. [57]	✓	✓	✓	✓	x	x
Verhagen et al. [63]	✓	✓	✓	✓	✓	✓
Wedderkopp et al. [58]	✓	x	✓	x	x	x
Zarei et al. [59]	✓	✓	✓	✓	✓	x
Zarei et al. [60]	✓	✓	✓	x	x	x
Zouita et al. [61]	✓	x	x	x	x	x

Articles that included strength, plyometric, stability, speed/agility, coordination/warm-up drills, and flexibility components were assessed to determine if they met training prescription recommendations to reach an effective stimulus. ✓ symbols in a green cell indicate the article met the muscular strength (included at least 2 sets, 8–15 repetitions or 20–30 s duration AND one progression (i.e. increased intensity/difficulty)) [22], plyometric (included at least 1 set, 3–15 repetitions or 10–30 s duration, AND one progression (i.e. increased intensity/difficulty)) [22], stability (included at least 240 s of stability training per session AND one progression (i.e. increased intensity/difficulty)) [36], speed/agility (included sprint-specific training exercises (unresisted/resisted forward and backward running with or without change of direction) between 10 and 30 m of distance) [37], coordination/warm-up drills (included at least 180 s of warm-up drills (e.g. skipping, heel flicks)) [38], and flexibility (included at least 1 stretch, 30 s duration (static) or 15 reps (dynamic/ballistic techniques) per session) [39] recommendations, while ✓ symbols in an orange cell indicate they did not meet these recommendations (or did not provide information about volume/intensity). x symbols in a red cell indicate that the intervention did not include this component. Only those components marked with ✓ symbols in green cells were considered when the restrictive approach was applied.

### 3.3. Quality and risk of bias assessments

Regarding the reporting quality of the studies, the mean score obtained with the CONSORT quality scale was 22.2 (minimum: 10, maximum: 29). According to the PEDro scale, the methodological quality of the studies showed a wide range of scores [2–9] with an average score of 5.8. Not all studies that were included in this review were randomized controlled designs and subsequently had lower quality scores. Regarding the risk of bias, the mean score obtained was 8 (minimum: 3, maximum: 11). The detailed data for CONSORT, PEDro, and risk of bias are presented in online supplementary files 9, 10, and 11, respectively.

### 3.4. Inference

#### 3.4.1. Pooled effects of IPPs on reducing overall and some specific body region injury incidences (first level of concretion)

The pooled IRR for each type of injury and its 95% confidence and prediction intervals, alongside the between-study variance, the *Q* test, and the *I^2^* index are displayed in Table 3. The combined estimates for the different dependent variables were very close (from 0.61 to 0.712) and all of them were significantly different from 1, which indicates evidence in favor of the IPP group over the control group.

**Table 3.** Pooled estimate, 95% confidence interval, and heterogeneity statistics for the dependent variables of the study.

Injuries	k	*N*	${\text{IRR}}^{+}$	95%CI	95%PI	$\tau^{2}$	$I^{2}$	Q	*p*
Total	21	18305	0.624	[0.538, 0.725]	[0.352, 1.108]	0.070	75.58	80.08	<0.001
Lower limbs	17	13898	0.634	[0.541, 0.743]	[0.382, 1.054]	0.052	62.30	44.18	<0.001
Thighs	13	11425	0.712	[0.524, 0.968]	[0.440, 1.151]	0.029	11.45	13.89	0.308
Knees	13	11425	0.659	[0.499, 0.871]	[0.342, 1.271]	0.074	40.73	18.71	0.096
Ankles	13	11425	0.610	[0.431, 0.862]	[0.228, 1.633]	0.179	62.87	29.51	0.003

*k* = number of independent samples included in the analysis; *N* = total sample size; ${\text{IRR}}^{+}$ = pooled effect size estimate (Incidence rate ratio); 95%CI = lower and upper bounds of the 95% confidence interval; 95%PI = lower and upper bounds of the 95% prediction interval; $\tau^{2}$ =Paule-Mandel estimate for the between-study variance; $I^{2}$ = heterogeneity index; *Q* = Cochran's heterogeneity statistic with k−1 degrees of freedom; *p* =* p*-value for the Cochran's heterogeneity statistic.

Unlike thigh and knee pooled IRRs, evidence of heterogeneity was found for the overall, lower extremity, and ankle pooled IRRs, as they showed statistically significant *Q* scores, large *I^2^* indexes (ranging from 62.3 to 75.6%), and wide prediction intervals including a range of values that would cast doubts on the overall beneficial effect of IPPs. Figure 2 shows the forest plot generated for each dependent variable meta-analyzed under a random-effects model.

Figure 2.Forest plots for the random-effects classical meta-analyses conducted for overall, lower extremity, thigh, knee, and ankle injuries. Values below 1 favor the interventions over the control groups.

**Figure:**
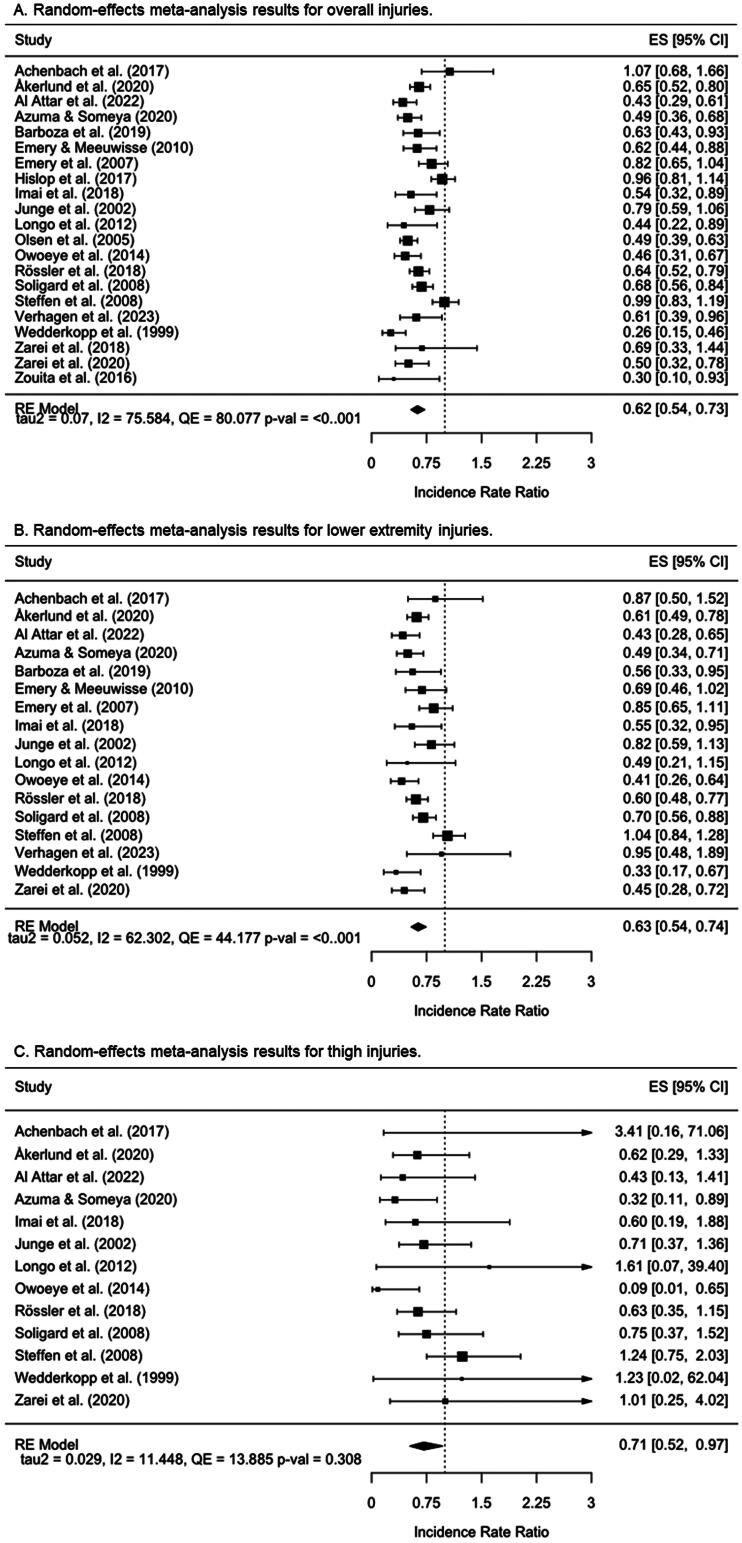

**Figure:**
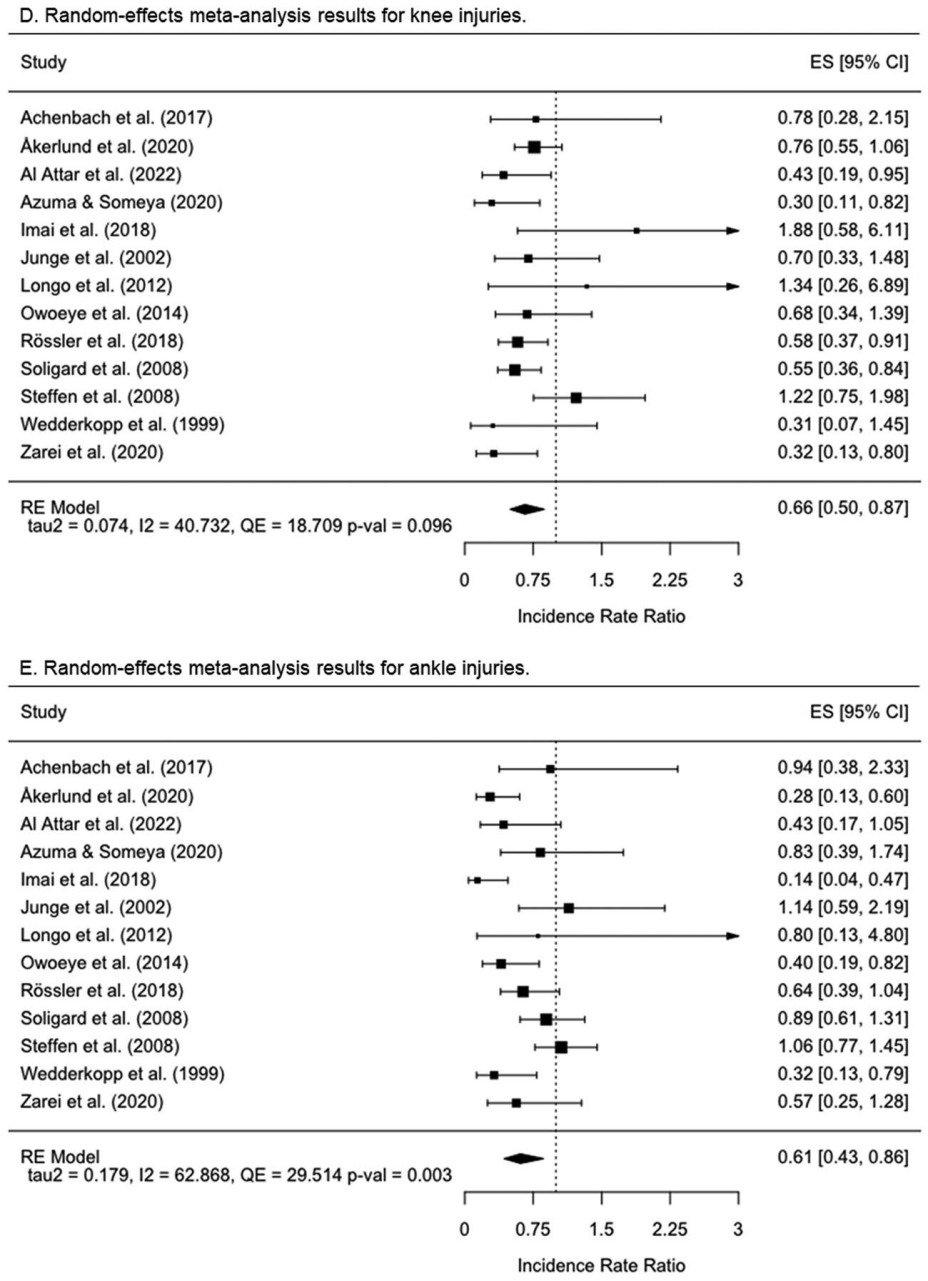

Regarding the analysis of potential qualitative moderators, supplementary file 12 presents the results of the ANOVAs conducted in each dependent variable. None of the categorical moderators showed a statistically significant relationship with the overall injury IRR (*p*-values for the Knapp-Hartung *F* statistics ranged from 0.747. to 0.912). Supplementary file 13 shows the results of the simple meta-regressions applied to each type of injury. None of the quantitative moderators analyzed showed a statistically significant relationship with the dependent variable overall injuries (*p* > 0.05).

Funnel plots and Egger's regression test results are presented in supplementary file 14. For overall, lower extremity and ankle injuries, Egger's regression coefficient estimates were statistically different from zero (*p*-values ranging from 0.006 and 0.047), which may indicate a possible overestimation in the meta-analytic results due to publication bias, unlike for thigh (*p* = 0.42) and knee (*p* = 0.59) injuries.

#### 3.4.2. Multiple comparisons between the injury risk mitigation pooled effects of the different IPPs and with the control group (second level of concretion)

##### 3.4.2.1. Overall injuries

**Figure 3.**
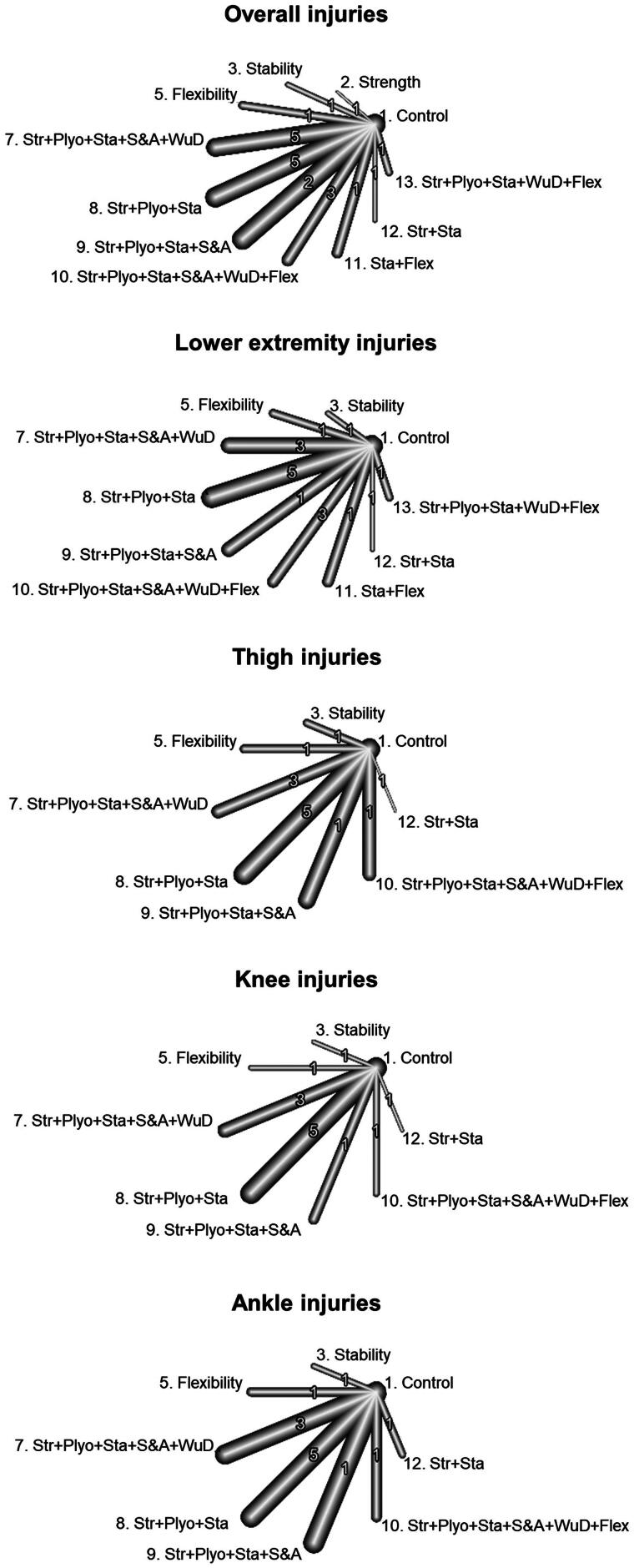
Network graphs for the direct evidence comparing programs for overall, lower extremity, thigh, knee, and ankle injuries under the comprehensive approach. Str: Strength; Plyo: Plyometrics; Sta: Stability; S&A: Speed and agility; WuD: Coordination and warm-up drills; Flex: Flexibility.

**Figure 4.**
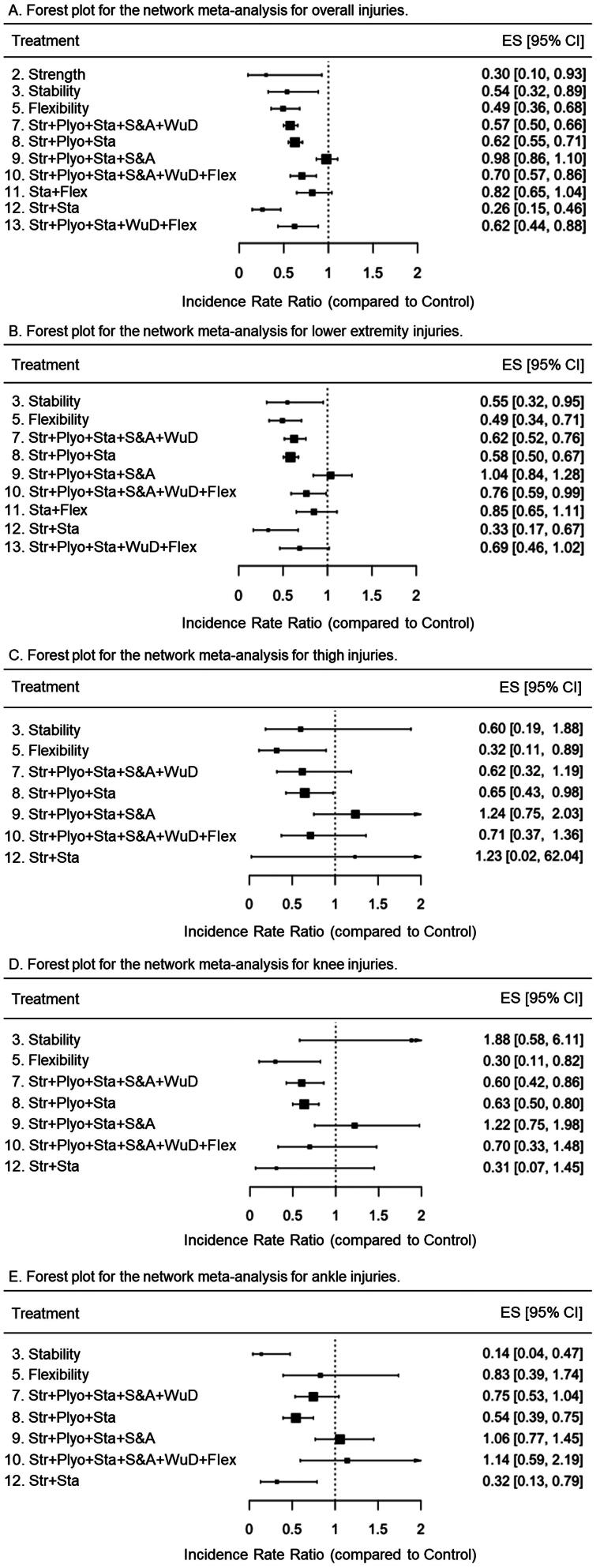
Forest plots for the random-effects network meta-analyses conducted for overall, lower extremity, thigh, knee, and ankle injuries under the comprehensive approach. Str: Strength; Plyo: Plyometrics; Sta: Stability; S&A: Speed and agility; WuD: Coordination and warm-up drills; Flex: Flexibility. Values below 1 favor the intervention over the control (treatment 1).

The network graph built for the dependent variable overall injury (Figure 3a) under the comprehensive approach shows that all the IPPs (*n* = 10 [(labels 2, 3, 5, 7-13]) incorporated into the network meta-analysis were directly compared with the control group in the 21 primary studies included [11–14,19,20, 53–67], whereas only indirect evidence was available for comparisons between the remaining IPPs. Unlike the IPPs labeled as 9 (strength + plyometrics + stability + speed and agility) (IRR = 0.98 [95%CI = 0.86 to 1.1]) and 11 (stability + flexibility) (IRR = 0.82 [95%CI = 0.65 to 1.04]), the remaining IPPs were more effective than a control group to reduce the injury risk in youth team sport athletes (Figure 4a). The IPPs with the highest injury risk mitigation effects were those comprised of strength + stability exercises (M-IPP labeled as 12) (IRR = 0.26 [95%CI = 0.15 to 0.46]), strength exercises (S-IPP labeled as 2) (IRR = 0.3 [95%CI = 0.1 to 0.93]), and flexibility (S-IPP labeled as 5) (IRR = 0.49 [95%CI = 0.36 to 0.68]). Table 4 presents the network meta-analysis estimates for the 55 possible pair-wise comparisons between programs.

**Table 4.** Results of the network meta-analysis under the comprehensive approach for overall injuries: Estimates and 95% confidence intervals for comparisons between each pair of injury prevention programs (programs in rows vs. programs in columns).

Program											
1	1										
2	0.302 [0.098, 0.925]	2									
3	0.536 [0.325, 0.886]	1.778 [0.521, 6.071]	3								
5	0.492 [0.357, 0.677]	1.63 [0.508, 5.228]	0.917 [0.506, 1.663]	5							
7	0.571 [0.497, 0.656]	1.892 [0.612, 5.851]	1.064 [0.632, 1.791]	1.16 [0.819, 1.644]	7						
8	0.625 [0.551, 0.709]	2.071 [0.67, 6.396]	1.165 [0.694, 1.955]	1.27 [0.901, 1.791]	1.095 [0.908, 1.32]	8					
9	0.977 [0.865, 1.104]	3.238 [1.049, 9.996]	1.821 [1.086, 3.053]	1.986 [1.411, 2.796]	1.712 [1.423, 2.058]	1.564 [1.312, 1.864]	9				
10	0.703 [0.572, 0.864]	2.33 [0.746, 7.28]	1.31 [0.762, 2.255]	1.429 [0.977, 2.09]	1.232 [0.961, 1.578]	1.125 [0.884, 1.432]	0.72 [0.566, 0.914]	10			
11	0.819 [0.645, 1.039]	2.713 [0.863, 8.531]	1.526 [0.875, 2.66]	1.664 [1.117, 2.479]	1.434 [1.089, 1.889]	1.31 [1, 1.716]	0.838 [0.641, 1.095]	1.164 [0.85, 1.595]	11		
12	0.261 [0.147, 0.465]	0.865 [0.245, 3.052]	0.487 [0.227, 1.046]	0.531 [0.275, 1.026]	0.457 [0.253, 0.828]	0.418 [0.232, 0.754]	0.267 [0.148, 0.482]	0.371 [0.201, 0.685]	0.319 [0.171, 0.595]	12	
13	0.621 [0.436, 0.885]	2.058 [0.635, 6.666]	1.157 [0.626, 2.14]	1.262 [0.784, 2.034]	1.088 [0.744, 1.591]	0.994 [0.682, 1.447]	0.636 [0.437, 0.924]	0.883 [0.586, 1.33]	0.759 [0.495, 1.162]	2.378 [1.209, 4.679]	13

*Note*. Program 1 = Control; Program 2 = Strength; Program 3 = Stability; Program 5 = Flexibility; Program 7 = Strength + plyometric + stability + speed & agility + warm-up drills; Program 8 = Strength + plyometric + stability; Program 9 = Strength + plyometric + stability + speed & agility; Program 10 = Strength + plyometric + stability + speed & agility + warm-up drills + flexibility; Program 11 = Stability + flexibility; Program 12 = Strength + stability; Program 13 = Strength + plyometric + stability + warm-up drills + flexibility. Values below 1 favor the row intervention (IPP).

**Figure 5.**
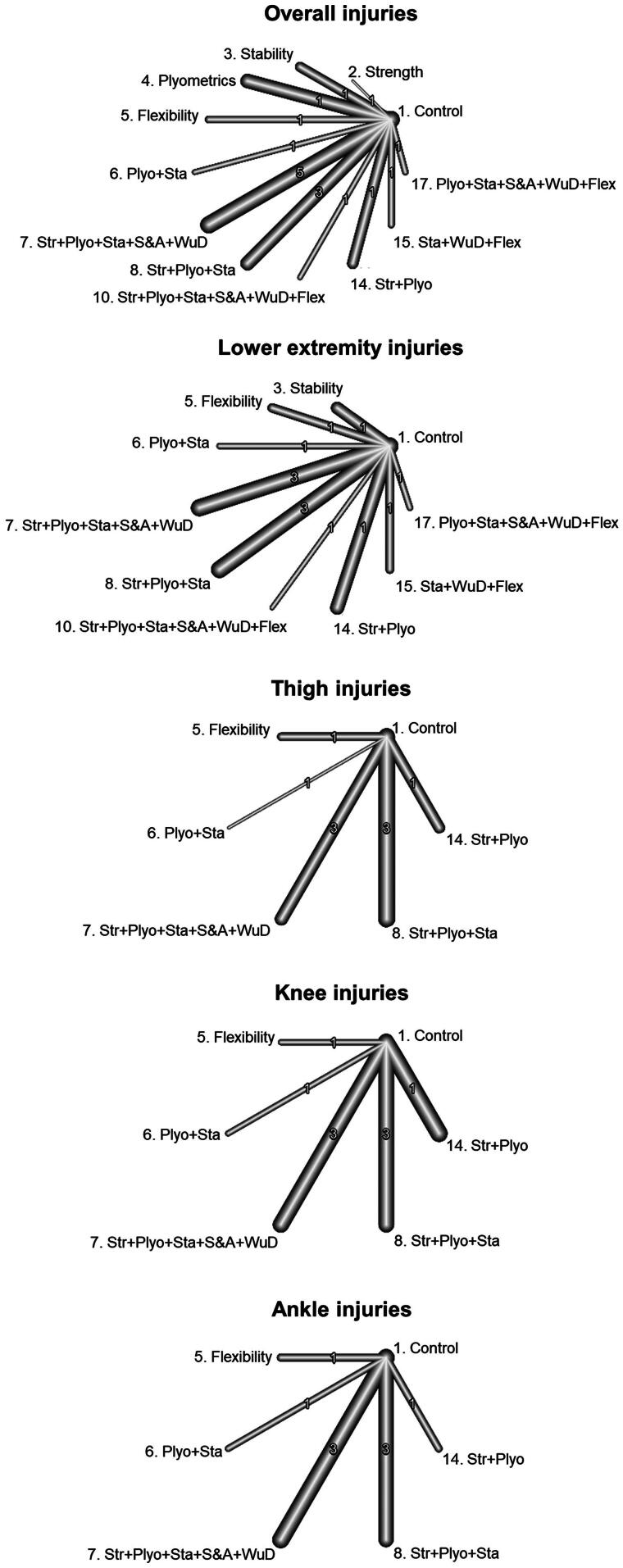
Network graphs for the direct evidence comparing programs for overall, lower extremity, thigh, knee, and ankle injuries under the restrictive approach. Str: Strength; Plyo: Plyometrics; Sta: Stability; S&A: Speed and agility; WuD: Coordination and warm-up drills; Flex: Flexibility.

**Figure 6.**
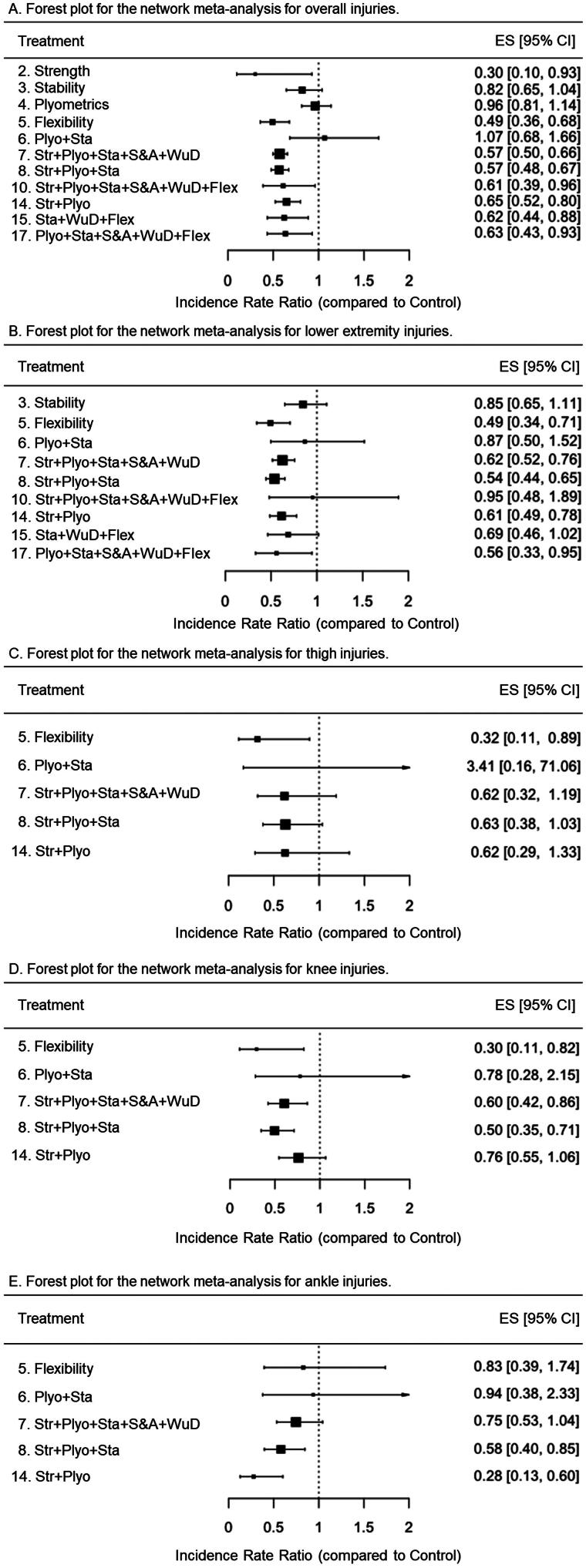
Forest plots for the random-effects network meta-analyses conducted for overall, lower extremity, thigh, knee, and ankle injuries under the restrictive approach. Str: Strength; Plyo: Plyometrics; Sta: Stability; S&A: Speed and agility; WuD: Coordination and warm-up drills; Flex: Flexibility. Values below 1 favor the intervention over the control (treatment 1).

When the restrictive approach was applied to determine whether a single exercise component should be integrated into an IPP, the network meta-analysis comparing the protective effects of the IPPs (*n* = 11) (Figure 5a) with control groups through 17 primary studies [11–14,19,54–56,59–67] revealed that most IPPs (except those labeled 3 [stability], 4 [plyometrics], and 6 [plyometrics + stability]) were more effective than control groups at reducing the overall incidence of injuries in youth team sport athletes (Figure 6a). Similarly to the findings of the comprehensive approach, IPP programs with the highest protective effects were those consisting of strength exercises (S-IPP labeled as 2) (IRR = 0.3 [95%CI = 0.1 to 0.93]) and flexibility exercises (S-IPP labeled as 5) (IRR = 0.49 [95%CI = 0.36 to 0.68]), followed by a set of M-IPPs with almost identical protective effects (IRRs ranged from 0.57 to 0.65), led by those labeled as 7 (strength + plyometrics + stability + speed & agility + warm-up drills) and 8 (strength + plyometrics + stability). Table 5 presents the network meta-analysis estimates for the 66 possible pair-wise comparisons between programs.

**Table 5.** Results of the network meta-analysis under the restrictive approach for overall injuries: Estimates and 95% confidence intervals for comparisons between each pair of programs (programs in rows vs. programs in columns).

Program												
1	1											
2	0.302 [0.098, 0.925]	2										
3	0.819 [0.645, 1.039]	2.713 [0.863, 8.531]	3									
4	0.963 [0.815, 1.138]	3.191 [1.028, 9.908]	1.176 [0.879, 1.573]	4								
5	0.492 [0.357, 0.677]	1.63 [0.508, 5.228]	0.601 [0.403, 0.895]	0.511 [0.356, 0.733]	5							
6	1.066 [0.684, 1.661]	3.533 [1.058, 11.792]	1.302 [0.787, 2.155]	1.107 [0.689, 1.779]	2.167 [1.254, 3.744]	6						
7	0.571 [0.497, 0.656]	1.892 [0.612, 5.851]	0.697 [0.529, 0.919]	0.593 [0.477, 0.736]	1.16 [0.819, 1.644]	0.535 [0.336, 0.852]	7					
8	0.566 [0.479, 0.67]	1.876 [0.604, 5.826]	0.692 [0.517, 0.926]	0.588 [0.464, 0.745]	1.151 [0.802, 1.651]	0.531 [0.33, 0.853]	0.992 [0.798, 1.233]	8				
10	0.61 [0.387, 0.96]	2.02 [0.603, 6.768]	0.745 [0.446, 1.243]	0.633 [0.391, 1.027]	1.239 [0.712, 2.159]	0.572 [0.303, 1.079]	1.068 [0.665, 1.716]	1.077 [0.664, 1.747]	10			
14	0.647 [0.524, 0.8]	2.144 [0.685, 6.708]	0.79 [0.575, 1.087]	0.672 [0.513, 0.88]	1.315 [0.897, 1.93]	0.607 [0.371, 0.992]	1.134 [0.88, 1.46]	1.143 [0.872, 1.498]	1.061 [0.643, 1.751]	14		
15	0.621 [0.436, 0.885]	2.058 [0.635, 6.666]	0.759 [0.495, 1.162]	0.645 [0.436, 0.954]	1.262 [0.784, 2.034]	0.582 [0.33, 1.028]	1.088 [0.744, 1.591]	1.097 [0.741, 1.623]	1.019 [0.573, 1.811]	0.96 [0.635, 1.45]	15	
17	0.635 [0.434, 0.928]	2.103 [0.644, 6.868]	0.775 [0.495, 1.214]	0.659 [0.435, 0.998]	1.29 [0.785, 2.12]	0.595 [0.332, 1.068]	1.112 [0.742, 1.666]	1.121 [0.74, 1.699]	1.041 [0.576, 1.882]	0.981 [0.635, 1.516]	1.022 [0.608, 1.719]	17

*Note.* Program 1 = Control; Program 2 = Strength; Program 3 = Stability; Program 4 = Plyometrics; Program 5 = Flexibility; Program 6 = Plyometrics + stability; Program 7 = Strength + plyometrics + stability + speed & agility + drills; Program 8 = Strength + plyometrics + stability; Program 10 = Strength + plyometrics + stability + speed & agility + drills + flexibility; Program 14 = Strength + plyometrics; Program 15 = Stability + drills + flexibility; Program 17 = Plyometrics + stability + speed & agility + drills + flexibility. Values below 1 favor the row intervention.

##### 3.4.2.2. Lower extremity injuries

A total of nine IPPs (labeled 3, 5, 7–13) were included in the network meta-analysis to make direct comparisons between their effects on lower extremity IIRs and those elicited by control groups, using 17 primary studies [11–14,19,20,53,54,56–58,60,62–66] (Figure 3b). Only indirect evidence was available for comparisons between the remaining IPPs in this network meta-analysis. All programs except three M-IPPs (labeled 9 [strength + plyometrics + stability + speed and agility], 11 [stability + flexibility], and 13 [strength + plyometrics + stability + warm-up drills + flexibility]) were found to be more effective than the control groups at reducing lower extremity IIRs (Figure 4b). As shown in supplementary file 15, the IPP labeled as 12 (strength + stability) elicited the highest risk mitigation effects for lower extremity injuries (IRR = 0.33 [95%CI = 0.17 to 0.67]). Supplementary file 15 presents the network meta-analysis estimates for the 45 possible pair-wise comparisons between programs.

When the restrictive approach was applied, the network meta-analysis directly compared nine IPPs (labeled 3, 5–8, 10, 14, 15, 17) and the control group using 13 primary studies [11–14,19,54,56,60,62–66] (Figure 5b). However, only indirect evidence was available for comparisons between the remaining programs. Unlike the IPPs labeled as 3 (stability) (IRR = 0.85 [95%CI = 0.65 to 1.11]), 6 (plyometrics + stability) (IRR = 0.87 [95%CI = 0.50 to 1.52]), 10 (strength + plyometrics + stability + speed and agility + warm-up drills + flexibility) (IRR = 0.95 [95%CI = 0.48 to 1.89]), and 15 (stability + warm-up drills + flexibility) (IRR = 0.69 [95%CI = 0.46 to 1.02]), the paired comparisons conducted revealed that the remaining IPPs were more effective than a control group to reduce the lower extremity injury risk in youth team sport athletes. The IPP labeled as 5 (flexibility) elicited the highest risk mitigation effects for lower extremity injuries (IRR = 0.49 [95%CI = 0.34 to 0.71]), followed by the labeled as 8 (strength + plyometrics + stability) (IRR = 0.54 [95%CI = 0.44 to 0.65]), and 17 (plyometrics + stability + speed and agility + warm-up drills + flexibility) (IRR = 0.56 [95%CI = 0.33 to 0.95]) (Figure 6b). Supplementary file 16 presents the network meta-analysis estimates for the 45 possible pair-wise comparisons between programs.

##### 3.4.2.3. Thigh injuries

The network graph presented in Figure 3c shows that seven IPPs (labels 3, 5, 7-10, 12) included in the network meta-analysis were directly compared with the control group in the 13 primary studies incorporated [11,12,19,20,53,54,56–58,60,62,64,65], whereas only indirect evidence was available for comparisons between the remaining IPPs. Only the S-IPP number 5 (flexibility) (IRR = 0.32 [95%CI = 0.11 to 0.89]) and the M-IPP number 8 (strength + plyometrics + stability) (IRR = 0.65 [95%CI = 0.43 to 0.98]) were more effective than the control group to reduce the thigh IIRs (Figure 4c). In the supplementary file 15, it can be found the network meta-analysis estimates for the 28 possible pair-wise comparisons between IPPs.

When the restrictive approach was applied, the network meta-analysis directly compared five IPPs (labeled 5–8, 14) and the control group using nine primary studies [11,12,19,54,56,60,62,64,65]. However, only indirect evidence was available for comparisons between the remaining programs. Similar to what was found under the comprehensive approach, just the S-IPP number 5 (flexibility) was more effective than the control group to reduce the thigh IIRs (IRR = 0.32 [95%CI = 0.11 to 0.89]) (Figure 6c). In the supplementary file 16, it can be found the network meta-analysis estimates for the 15 possible pair-wise comparisons between IPPs.

##### 3.4.2.4. Knee injuries

The network graph presented in Figure 3d shows that seven IPPs (labels 3, 5, 7-10, 12) included in the knee injury network meta-analysis were directly compared with the control group in the 13 primary studies incorporated [11,12,19,20,53,54,56–58,60,62,64,65], whereas only indirect evidence was available for comparisons between the remaining IPPs. Only the IPPs numbers 5 (flexibility) (IRR = 0.30 [95%CI = 0.11 to 0.82]), 7 (strength + plyometrics + stability + speed & agility + warm-up drills) (IRR = 0.60 [95%CI = 0.42 to 0.86]), and 8 (strength + plyometrics + stability) (IRR = 0.63 [95%CI = 0.50 to 0.80]) were more effective than the control group to reduce the knee IIRs in youth team sport athletes (Figure 4d). Supplementary file 15 presents the network meta-analysis estimates for the 28 possible pair-wise comparisons between programs and their confidence interval bounds.

When the restrictive approach was applied, the network meta-analysis directly compared five IPPs (labeled 5–8, 14) and the control group using 9 primary studies [11,12,19,54,56,60,62,64,65], while only indirect evidence was available for comparisons between the remaining programs. Unlike the IPPs labeled as 6 (plyometrics + stability) (IRR = 0.78 [95%CI = 0.28 to 2.15]) and 14 (strength + plyometrics) (IRR = 0.76 [95%CI = 0.55 to 1.06]), the paired comparisons conducted revealed that the remaining three IPPs were more effective than a control group to reduce the lower extremity injury risk in youth team sport athletes (Figure 6d). Again, the IPP with the lowest IRR was the number 5 (flexibility) (IRR = 0.30 [95%CI = 0.11 to 0.82]). In the supplementary file 16, it can be found the network meta-analysis estimates for the 15 possible pair-wise comparisons between IPPs.

##### 3.4.2.5. Ankle injuries

Seven IPPs (labels 3, 5, 7-10, 12) were included in the ankle injury network meta-analysis (Figure 3e) and were directly compared with the control group in the 13 primary studies included [11,12,19,20,53,54,56–58,60,62,64,65], whereas only indirect evidence was available for comparisons between the remaining programs. Three out of the seven IPPs that were incorporated into the network meta-analysis were more effective than the control group to reduce ankle IIRs (IPPs number 3 [stability], 8 [strength + plyometrics + stability], and 12 [strength + stability]), being the S-IPP labeled as 3 the one with the lowest IRR (0.14 [95%CI = 0.04 to 0.47]) (Figure 4e). Supplementary file 15 presents the network meta-analysis estimates for the 28 possible pair-wise comparisons between programs.

Under the restrictive approach, the network meta-analysis directly compared five IPPs (labeled 5–8, 14) and the control group using nine primary studies [11,12,19,54,56,60,62,64,65]. However, only indirect evidence was available for comparison for the remaining programs. Two M-IPPs labeled as 14 (strength + plyometrics) (IRR = 0.28 [95%CI = 0.13 to 0.60]) and 8 (strength + plyometrics + stability) (IRR = 0.58 [95%CI = 0.40 to 0.85]) were more effective than the control group to reduce ankle IIRs. In the supplementary file 16, it can be found the network meta-analysis estimates for the 15 possible pair-wise comparisons between IPPs.

#### 3.4.3. Individual effects of exercise-based components (third level of concretion)

##### 3.4.3.1. Overall injuries

**Figure 7.**
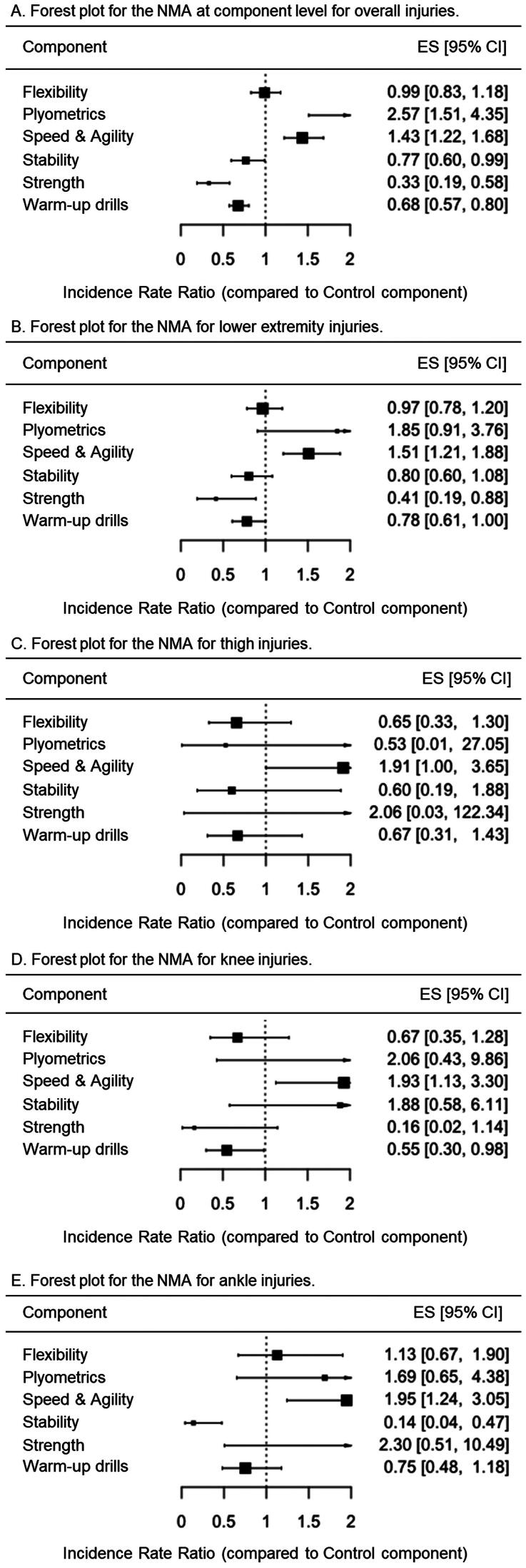
Forest plots for the random-effects network meta-analyses at component level conducted for overall, lower extremity, thigh, knee, and ankle injuries under the comprehensive approach. Values below 1 favor the training component over the control.

**Figure 8.**
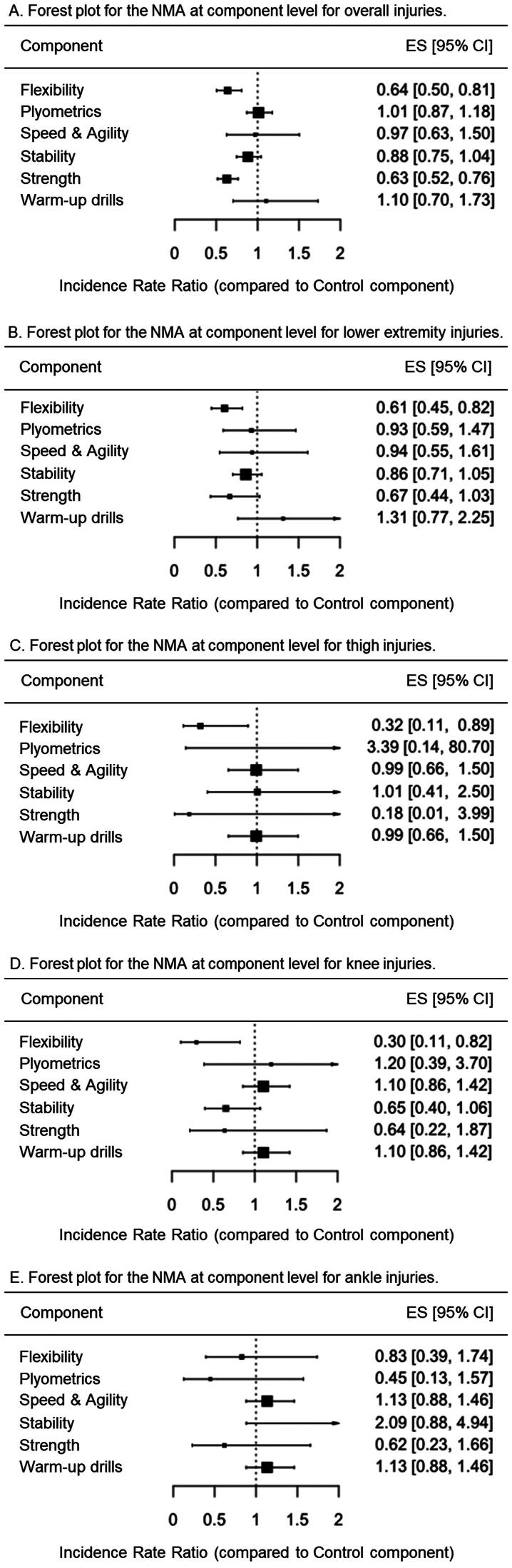
Forest plots for the random-effects network meta-analyses at component level conducted for overall, lower extremity, thigh, knee, and ankle injuries under the restrictive approach. Values below 1 favor the training component over the control.

As shown in Figure 7a, the network meta-analysis at the component level, conducted using a comprehensive approach, revealed that three exercise-based components demonstrated significant injury risk mitigation effects on overall IIRs: strength (IRR = 0.33 [95%CI = 0.19 to 0.58]), warm-up drills (IRR = 0.68 [95%CI = 0.57 to 0.80]) and stability (IRR = 0.77 [95%CI = 0.60 to 0.99]). On the contrary, neither flexibility (IRR = 0.99 [95%CI = 0.83 to 1.18]), speed and agility (IRR = 1.43 [95%CI = 1.22 to 1.68]) nor plyometric (IRR = 2.57 [95%CI = 1.51 to 4.35]) components reported statistically significant effects on overall IIRs. However, when the analysis was conducted using a restrictive approach, only strength (IRR = 0.63 [95%CI = 0.52 to 0.76]) and flexibility (IRR = 0.64 [95%CI = 0.50 to 0.81]) as exercise-based components reported significant injury risk mitigation effects on overall IIRs (Figure 8a).

##### 3.4.3.2. Lower extremity injuries

The network meta-analysis at the component level conducted with lower extremity injuries as the dependent variable and using the comprehensive approach showed that only the exercise-based components strength (IRR = 0.41 [95%CI = 0.19 to 0.88]) presented significant reduction effects on their incidence rates (Figure 7b). When the analysis was conducted under the restrictive approach, flexibility (IRR = 0.61 [95%CI = 0.45 to 0.82]) was the only component reporting significant injury risk mitigation effects on lower extremity IIRs (Figure 8b).

##### 3.4.3.3. Thigh injuries

For thigh injuries, the network meta-analysis at component level conducted using the comprehensive approach reported that none of the exercise-based components showed significant reduction effects on their IIRs (Figure 7c). However, under the restrictive approach, the flexibility (IRR = 0.32 [95%CI = 0.11 to 0.89]) demonstrated significant injury risk mitigation effects on thigh IIRs (Figure 8c).

##### 3.4.3.4. Knee injuries

For its part, only the exercise-based components warm-up drills for knee (IRR = 0.55 [95%CI = 0.30 to 0.98]) elicited significant reduction effects for these IIRs under the comprehensive approach (Figure 7d). On the contrary, when the restrictive analysis was carried out, the component flexibility (IRR = 0.30 [95%CI = 0.11 to 0.82]) was the only one showing significant reduction effects on knee IIRs (Figure 8d).

##### 3.4.3.5. Ankle injuries

For ankle injuries, only the stability component (IRR = 0.14 [95%CI = 0.04 to 0.47]) showed significant reduction effects for IIRs under the comprehensive approach (Figure 7e). No component demonstrated a significant reduction on their IIRs for the restrictive analysis (Figure 8e).

## 4. Discussion

The primary purposes of this systematic review and meta-analysis were (a) to estimate the pooled effects of IPPs on reducing overall and some specific body regions injury incidences, and (b) to compare the effects of S-IPPs and M-IPPs on mitigating injury risk in youth team sport athletes. A secondary objective was to explore the individual effects of different exercise-based components (i.e. strength, plyometrics, stability, speed & agility, coordination/warm-up drills, and flexibility) on the injury incidences previously mentioned. To address these objectives, data extracted from 21 eligible studies were analyzed according to three different levels of concretion. Next, the main findings obtained from these three levels of analysis are presented and discussed separately.

### 4.1. Pooled effects of IPPs on reducing overall and some specific body region injury incidences

The results of the random-effects model classical meta-analysis conducted at this level of concretion indicate that exercise-based strategies elicit statistically significant protective effects against the injuries sustained by youth team sport athletes. Specifically, IPPs may reduce overall and some specific body regions (lower extremities, thigh, knee, and ankle) IIRs by an average of approximately 35% (range 29–39%). The magnitude of the just mentioned protective effects of exercise-based strategies against injuries is analogous to those reported in previous meta-analyses carried out in team sports players with [17,18] and without [22,68] participants' age restriction. However, it should be pointed out that most of the studies included in the present meta-analysis (12 out of 21) and also in previously published research [17,18,33] recruited youth soccer players as participants. Therefore, future studies are required to elucidate whether the risk mitigation effects we found for IPPs might be extrapolated to other youth team sports athletes different from soccer players. Considering the high risk of injury documented in previous epidemiological studies for a number of different youth team sports [4–6] along with the protective effects against them estimated in this systematic review and meta-analysis for the selected exercise-based strategies, it may be stated that implementing these effective and timely IPPs in applied contexts is advisable.

Exploring potential sources of heterogeneity (moderator analysis) of the pooled estimates of the effects of IPPs could shed light on the factors that may modulate them. In this regard, the results from the quantitative moderator analysis (meta-regressions) carried out in this systematic review and meta-analysis show that the high values of the *I^2^* statistic for the pooled IRRs of IPPs for overall, lower extremity, knee, and ankle injuries (supplementary file 17) cannot be explained by the large heterogeneity of the scores obtained by individual studies on the different quality scales (of the information provided and methodology used) and risk of bias tools (CONSORT [*p*-values > 0.33], PEDro [*p*-values > 0.49], and risk of bias [*p*-values > 0.25]) applied. It must be acknowledged that this conclusion is exclusively limited to the total scores from the quality scales and risk of bias tool applied in this systematic review to the selected studies. These results are consistent with the findings reported by Soomro et al. [17] in their meta-analysis on the efficacy of injury prevention programs in youth team sports, where it was documented that the quality of the studies (determined through an earlier version of our risk of bias scale) did not have a statistically significant effect on the magnitude of the pooled effect of the IPPs. Future studies that carry out more in-depth qualitative and quantitative analyses to determine the individual impact of the scores obtained by the studies in each domain of the quality scales and risk of bias tools on the pooled IRR of each type of injury could improve current knowledge about whether there is a relationship (including its direction and magnitude) between these factors and the risk-mitigating effects of the IPPs observed in them.

The analysis of quantitative moderators also indicates that the male rate did not affect the IRRs for overall injuries. These results are in line with the recent findings of a meta-analysis analyzing the effect of sex on overall injury prevention effectiveness in sport [69]. IPPs should then be implemented to reduce overall injuries in youth environments, irrespective of the sex of the participants. However, when analyzed by specific body regions, it can be observed that the male rate accounted for 41.1% and 100% of the variance of the IRRs for the lower extremity and thigh injuries (*p* values for the *F* statistics < 0.05). These results would suggest that, for reducing lower extremity and thigh injuries, IPPs are favored over the control group to a greater extent in those studies where the male rate was lower. Given that male team-sport athletes have a higher incidence of injuries in this region than females [70], this might be considered an important finding as it could be indicating that the interventions analyzed may not be sufficient to reduce the incidence of thigh injuries among male players. However, it should be noted that there is a large imbalance between the number of trials that have used only males as a sample of study (*n* = 10) and those that have exclusively recruited females (*n* = 3), which makes it difficult to draw strong conclusions in this regard.

The results from the meta-regressions also reveal that neither the length (weeks) nor the duration (minutes) of IPPs had a statistically significant influence on the overall injury risk mitigation effects. However, unlike the length, the duration of each IPP accounted for 2.7%, 17.8%, and 50.4% of the variance of the lower extremity, knee, and ankle injuries respectively, although this moderator only reached statistically significant results in the case of the ankle injuries (*p*-value = 0.05). In other words, there is little evidence pointing out that the duration of IPPs might play a significant role in their protective effects, which requires further studies to be elucidated. The minimum length and duration of IPPs included in this systematic review and meta-analysis were 12 weeks and 15 min, respectively. Consequently, it might be recommended that this was the minimum dose necessary for their protective effects against injuries to be relevant. Most of the studies included in this systematic review and meta-analysis used a weekly frequency of 2 days and hence, it was not possible to infer whether this moderating variable had an impact on the pooled IRRs of the IPPs. Likewise, and as in previous meta-analyses [17,18], the limited data availability (less than 50% of the studies did report any type of compliance) together with the different definitions and methods used [71] prevented us from analyzing the compliance with the IPPs as a potential moderator in this research. Nevertheless, previous studies implementing the same program (i.e. 11+), and within the same study context (female adolescent soccer players) and similar definition of the term compliance (i.e. player compliance), have shown an association between an increased individual player compliance to the IPP and an overall injury reduction effect [72,73]. It is therefore important for coaches to be aware of the need to comply with the program prescriptions. Future investigations should present operational definitions of this term so that it can be meta-analytically analyzed as well.

The qualitative moderator analyses (ANOVAs) carried out reveal that the equipment needed to implement the IPPs accounted for an important proportion of the variance of the following dependent variables: lower extremity (35.1%), thigh (100%), and knee (23.9%). The IRRs were, however, only significantly different (from a statistic standpoint) between those studies in which equipment was used and those in which it was not for lower extremity (*p* = 0.04) and thigh (*p* = 0.02) injuries. In both cases, the IPP groups seem to be favored over the control groups to a greater extent in those studies where the IPPs did not require any equipment. It should also be noted that the dichotomous moderator type of design (randomized *vs*. non-randomized) did not show a significant effect on the pooled IRRs of the dependent variables. One possible explanation for this could be based on the large imbalance that this moderator presents in the instances of its two response levels (RCT = 18 studies, N-RCT = 3 studies), which could have limited the ability of the ANOVA statistical technique to make a precise inference.

### 4.2. Multiple comparisons between the injury risk mitigation pooled effects of the different IPPs and with the control group

The direct pairwise comparisons (IPP *vs*. control group) carried out under both comprehensive and restrictive approaches show that two out of the four S-IPPs finally defined in this systematic review and meta-analysis (S-IPPs labeled as 2 [strength] and 5 [flexibility]) were more effective (IRRs = 0.30 [S-IPP number 2] and 0.49 [S-IPP number 5]) than their respective control groups (Figures 4 and 6) in reducing the overall IIRs in youth team sport athletes. Likewise, S-IPP number 3 (stability) was more effective than its respective control groups in reducing the risk of injury under the comprehensive approach (IRR = 0.54 [95%CI = 0.32 to 0.89) but not under the restrictive approach (IRR = 0.82 [95%CI = 0.65 to 1.04]), although the upper limit of the confidence interval was very close to 1 also for this restricted analysis. Whether the S-IPPs comprised of speed/agility exercises and coordination/warm-up drills are effective in reducing IRRs in team sports is still unknown as no studies have used them as isolated interventions in youth team sports.

The network meta-analysis conducted at this level of concretion also reported that most of the M-IPPs delivered by the selected studies demonstrated statistically significant risk mitigation effects for overall injuries (IRRs between 0.26 and 0.70) in youth team sport athletes regardless of the approach used for the analyses. However, the indirect paired comparisons performed between IPPs did not show a clear trend to recommend a specific intervention over other M-IPPs. The only program that could presumably reduce the risk of injury more than other interventions could be program 12 (strength + stability), but this hypothesis would only be supported by the comprehensive approach as this could not be analyzed from the restrictive method (components in this program did not meet the minimum training recommendations). These results are, however, in line with the IRRs provided in Tables 4 and 5 for S-IPPs, which might lead to suggest that, from a practical perspective, interventions comprised exclusively of strength (IRR = 0.3 [95%CI = 0.10 to 0.93]) (or mostly, as program 12) and flexibility (IRR = 0.49 [95%CI = 0.36 to 0.68]) exercises were the most effective measures (among all those included in this study) for reducing the number of injuries recorded in youth team sports. However, this statement should be considered with a degree of caution and serve as a starting point for future work, given that the effects on IIRs of each of these two S-IPPs came from a single regional study where a limited sample of soccer players (26 and 64 players for the S-IPPs based on strength and flexibility exercises, respectively) was recruited [19,61]. In contrast, the injury risk mitigation effects estimated for certain M-IPPs came from large-scale national [11,55,57,67] and multinational [12] studies carried out with a substantive number of participants. Therefore, taking into account the results of the paired comparisons (direct [IPPs *vs*. control] and indirect [IPPs *vs*. IPPs]) and the compiling of evidence available, it could be concluded that whether coaches and physical trainers seek to reduce the number of injuries in youth team sport athletes (around 40% on average), they may consider implementing in their training sessions the M-IPPs labeled with numbers 7 (strength + plyometrics + stability + speed & agility + warm-up drills), and 8 (strength + plyometrics + stability).

Finally, the results obtained in the pairwise comparisons carried out between the IIRs of the lower extremity, thigh, knee, and ankle of the different IPPs and with the control group (supplementary files 16 and 17) allow for similar conclusions to be drawn as previously exposed for the overall IIRs. However, the network meta-analysis carried out with thigh injuries as the dependent variable reports that out of the 7 (comprehensive approach) and 5 (restrictive approach) IPPs that were included to carry out direct and indirect comparisons, only the IPP comprised of flexibility exercises (S-IPP number 5) was more effective than its respective control groups to reduce the thigh IIRs (IRR = 0.32 [95%CI = 0.11 to 0.89]) in both approaches (Figures 4 and 6). Most of the injuries that occur in the thigh in youth team sports athletes are muscle strains (hamstrings, quadriceps, and adductors, mainly) [74–77]. In this type of injury, having adequate levels of flexibility may be considered a protective factor, since it contributes to optimizing the ability of the muscle-tendon unit to safely manage the high tensile forces to which it is subjected during the execution of the explosive locomotor actions (e.g. sudden accelerations and decelerations, changes of direction) that are repeatedly performed in intermittent team sports [19,78].

### 4.3. Individual effects of exercise-based components

The main findings from this third level of the analysis show that the exercise-based component strength was the only one that reported statistically significant injury risk mitigation effects on overall IIRs independently of the methodological approach used (comprehensive or restrictive) to calculate their estimations. This component was also associated with significant reduction of lower extremity injuries under the comprehensive approach and was very close to the statistical significance under the restrictive analysis (upper CI limit = 1.03). This is unsurprising as strength training increases the cross-sectional area and tensile strength of the musculotendinous system, while also increasing the rapid force production capability of the associated muscles. Increased rapid force production increases the ability to both rapidly accelerate and decelerate the athletes' mass, or their limb, based on the impulse momentum relationship. As such increased eccentric strength of the hamstrings has been shown to reduce hamstring strain injury risk and occurrence [79,80], while also improving landing mechanics which may reduce the risk of knee injuries [81]. Strength training plays an important role in reducing the risk of injuries related to muscle imbalance as well (either agonist/antagonist or side-to-side differences). Correction of the existing imbalances through this training component can mitigate the risk for musculoskeletal injury [61]. Adaptations resulting from strength training also include an increase in bone mineral density, which is particularly useful in collision sports (as is the case in most team sports) to reduce the risk of bone injuries [61,82].

Under the restrictive methodological approach, the exercise-based component of flexibility demonstrated statistically significant injury risk mitigation effects on overall (like the strength component) and lower extremity (unlike the rest of the components) IIRs. It has been suggested that poor values of muscle flexibility (especially in the biarticular muscle groups contributing to hip flexion [hamstrings], extension [quadriceps, psoas iliacus], abduction [adductors], and ankle dorsal flexion [gastrocnemius]) may have a negative impact not only on the ability of these soft tissue structures to withstand the high compressive and shear forces to which they are subjected during most of the explosive movements that team sport athletes repeatedly perform but also restrict normal joint ranges of motion [83]. Restrictions in joint ranges of motion may force athletes to adopt abnormal movement patterns during sports practice in which a sub-optimal distribution in certain joints of the mechanical stress generated during them could be produced, thereby increasing the likelihood of damaging (through both acute and overuse mechanisms) surrounding soft tissues [83]. Thus, for example, it has been suggested that, among other biomechanical adaptations, a lack of flexibility in the anterior thigh muscles (quadriceps and psoas iliacus) commonly carries a compensatory counterclockwise movement of the pelvis in the sagittal plane (anterior pelvic tilt) [83–85]. This anteriorly tilted pelvis position may affect to some extent the kinematics of the entire lower extremity during high-intensity weight-bearing actions, leading to the posterior thigh muscles originating in it (hamstrings) being in a sub-optimal length (overstretched) to perform (both concentrically and eccentrically), which might place them in a more vulnerable situation to be damaged [83–85]. In fact, recent studies employing contemporary statistical techniques from Machine Learning and Data Mining environments have identified flexibility as an important risk factor for injury in youth team sport athletes [86,87].

Another interesting finding extracted from this third level of analysis is the fact that the exercise components speed/agility and plyometrics did not show, under the restrictive approach, significant protective effects against injuries despite both being proposed as essential elements of any injury prevention and rehabilitation program due to their greater specificity regarding the mechanism that promotes some of the most frequently diagnosed injuries in this cohort of athletes (i.e. actions at high speed and with a prevalence of the stretch-shortening cycle [e.g. sudden accelerations and sprints, changes of direction, jumps, and falls]) [77]. Moreover, under the comprehensive approach, these two components even reported statistically significant effects in favor of the appearance of an injury (IRR = 1.43 [95%CI = 1.22 to 1.68] and 2.57 [95%CI = 1.51 to 4.35] for the speed/agility and plyometric components in overall injuries, respectively). Although the number of studies is reduced and therefore, it is not possible to make a solid positioning on these findings, it could be suggested that in this population cohort, where the training experience is still limited, the application of speed and plyometric exercises should be considered only in those youth athletes who demonstrate high levels of strength and sufficient motor competency to ensure the safe and adequate execution of the exercises that represent these components.

### 4.4. Limitations

Despite the strengths of the novel approach used in this research, allowing the simultaneous comparison of multiple programs and components within a single analysis even though most of these interventions have never been compared head-to-head in trials, there are some limitations that should be acknowledged. First, while the potential benefits of the IPPs seem to far outweigh the risks they could pose to youth athletes (all the 95%CIs indicated that the true estimate resided within the beneficial range for IPPs compared to control groups), the 95%PIs obtained for the five type of injuries analyzed showed upper limits whose values slightly exceed the value of 1. This suggests that the true effect for any one future study could still possibly fall beyond the beneficial range for IPPs; thus, practitioners should remain cautious when considering our findings to make decisions until further studies reduce uncertainty about these effects. Second, our taxonomy of interventions and control conditions was based on previous literature, piloting, and discussion in our team. Nevertheless, we acknowledge that the process of categorizing exercise-based components may be subjective and some exercises can be used for different purposes. Future work should agree on a process for node-making as well as agreeing on a taxonomy for classifying exercise-based components and interventions. Third, only indirect evidence was available for comparisons between interventions other than the control group, which means that the strength of inference made in a network meta-analysis is not as robust as it could be and that consistency between direct and indirect evidence could not be assessed. Moreover, three of the included studies were N-RCTs, which might have weakened the quality of evidence. Fourth, the training prescriptions used to conduct the restrictive analysis derived from general recommendations for the youth population and thus, they may not represent a sufficient stimulus to achieve adaptations in highly trained athletes. Likewise, the order of appearance of every single component in M-IPPs was not considered but just its presence. Further studies are needed to find the optimal dosage of exercise-based injury prevention programs and their components, with clear reporting on training dose. Finally, our reporting of injury incidence does not reflect consensus statements that suggest reporting training and match injuries separately [30]. However, we could not separate training and match injuries because most of the included studies did not report training and match injuries independently of each other for the five types of injury analyzed in this research. Likewise, other injury locations and types proposed in the consensus statements were not studied due to a lack of data available, nor was the effect of IPPs on injury burden. Other factors could not be examined owing to a lack of available or reported data, such as the effect of chronological and biological age, skill level, compliance rates, or type of sport. In fact and as already mentioned above, most of the studies included in this systematic review and meta-analysis have been focused on soccer players. The generalization of the results to other sports necessitates future studies. It should also be noted that, in accordance with the aim of the study, we have only analyzed exercise-based interventions in this work. Interventions exploring other aspects that could also play an important role on injury reduction (e.g. co-education, early specialization, sport technique) were beyond the scope of this work. New research should explore the effect of controlling for these factors on injury risk reduction.

## 5. Conclusions

Evidence coming from 21 different studies suggests that exercise-based injury prevention programs may reduce overall, lower extremity, thigh, knee, and ankle injuries in youth team sport athletes by 35%. Most of the S-IPPs and M-IPPs implemented in the included studies demonstrated statistically significant risk mitigation effects for overall and lower extremity injuries and thus, we advise against recommending standard practices or sham exercises (i.e. the usual control group in RCTs) as an injury prevention strategy. Our results also indicate that interventions comprising strength and flexibility exercises, and likely those incorporating stability exercises, might be the most effective measures for reducing injury incidence in youth team sports. However, this statement should be considered with caution and serve as a starting point for future work, given that only indirect evidence was available for comparisons between the intervention groups. Further investment in new trials that address the limitations of the current evidence and compare different IPPs is required to confirm these estimates with direct evidence.

## Supplementary Material

Supplemental Material

## Data Availability

R codes are publicly available on https://data.mendeley.com/datasets/njzj278dz3/2. Additional data that support the findings of this study are also available from the corresponding author, FA, upon reasonable request.
