# Supplementary material for: A systematic review and network meta-analysis on the effectiveness of exercise-based interventions for reducing the injury incidence in youth team-sport players. Part 1: an analysis by classical training components
Source: Ann Med. 2024 Oct 1;56(1):2408457. doi: 10.1080/07853890.2024.2408457 (PMC11445890; doi:10.1080/07853890.2024.2408457)
Supplement: Supplemental Material [file IANN_A_2408457_SM0607.zip › suppl_data/Supplementary file 11.docx]

| **Supplementary file 11.** Risk of bias assessment of the studies (Cochrane Back and Neck Group scale). | | | | | | | | | | | | | | |
| --- | --- | --- | --- | --- | --- | --- | --- | --- | --- | --- | --- | --- | --- | --- |
| **Reference** | **Criteria for assessing risk of bias** | | | | | | | | | | | | | **Score** |
|  | **1** | **2** | **3** | **4** | **5** | **6** | **7** | **8** | **9** | **10** | **11** | **12** | **13** |  |
| Achenbach et al. (2017) | Y | Y | N | Y | Y | Y | Y | Y | ? | ? | ? | Y | Y | 9 |
| Åkerlund et al. (2020) | Y | Y | N | N | Y | Y | Y | Y | N | ? | Y | Y | Y | 9 |
| Al Attar et al. (2023) | Y | Y | Y | Y | Y | Y | Y | Y | N | ? | Y | Y | Y | 11 |
| Azuma & Someya (2020) | Y | Y | N | N | N | Y | Y | Y | Y | ? | N | Y | Y | 8 |
| Barboza et al. (2019) | N | N | N | Y | Y | Y | Y | Y | Y | ? | Y | Y | N | 8 |
| Emery & Meeuwisse (2010) | Y | Y | Y | N | Y | Y | Y | Y | Y | ? | Y | Y | N | 10 |
| Emery et al. (2007) | Y | Y | N | Y | Y | Y | Y | Y | Y | ? | N | Y | N | 9 |
| Hislop et al. (2017) | Y | Y | Y | Y | N | Y | Y | Y | Y | ? | Y | Y | N | 10 |
| Imai et al. (2018) | N | N | N | N | ? | ? | Y | Y | ? | ? | ? | Y | N | 3 |
| Junge et al. (2002) | N | N | N | N | N | Y | Y | Y | Y | ? | ? | Y | N | 5 |
| Longo et al. (2012) | Y | Y | N | Y | Y | Y | Y | Y | N | ? | Y | Y | N | 9 |
| Olsen et al. (2005) | Y | Y | N | Y | Y | Y | Y | Y | Y | ? | N | Y | Y | 10 |
| Owoeye et al. (2014) | Y | Y | N | N | N | Y | Y | Y | Y | ? | N | Y | Y | 8 |
| Rössler et al. (2018) | Y | Y | N | N | Y | Y | Y | Y | ? | ? | N | Y | Y | 8 |
| Soligard et al. (2008) | Y | N | N | N | N | Y | Y | Y | ? | ? | Y | Y | Y | 7 |
| Steffen et al. (2008) | Y | Y | N | N | Y | Y | Y | Y | ? | ? | N | Y | Y | 8 |
| Verhagen et al. (2023) | Y | ? | N | N | N | Y | Y | Y | N | ? | N | Y | Y | 6 |
| Wedderkopp et al. (1999) | Y | N | N | N | N | Y | Y | Y | Y | N | ? | Y | Y | 7 |
| Zarei et al. (2018) | ? | ? | N | N | ? | Y | Y | Y | Y | ? | Y | Y | Y | 7 |
| Zarei et al. (2019) | Y | Y | N | N | Y | N | Y | Y | Y | ? | Y | Y | Y | 9 |
| Zouita et al. (2016) | Y | N | N | N | Y | ? | Y | Y | Y | ? | ? | Y | Y | 7 |
