# Supplementary material for: A systematic review and network meta-analysis on the effectiveness of exercise-based interventions for reducing the injury incidence in youth team-sport players. Part 1: an analysis by classical training components
Source: Ann Med. 2024 Oct 1;56(1):2408457. doi: 10.1080/07853890.2024.2408457 (PMC11445890; doi:10.1080/07853890.2024.2408457)
Supplement: Supplemental Material [file IANN_A_2408457_SM0607.zip › suppl_data/Supplementary file 12.docx]

| **Supplementary file 12.** Results of the weighted analyses of variance (ANOVAs) applied on the overall, lower extremity, thigh, knee, and ankle injuries estimates, taking qualitative moderator variables as independent variables. | | | | | | |
| --- | --- | --- | --- | --- | --- | --- |
| **Predictor** | ***k*** | **N** | $\boldsymbol{IRR}_{\boldsymbol{+}}$ | **LL** | **UL** | **ANOVA results** |
| *Overall injuries* | | | | | | |
| Design  Randomised  Non-randomised  Equipment  With  Without  Monitoring  Researcher  Trainer  Players | 18  3  7  13  1  14  3 | 17,746  559  6,328  9,525  194  13,559  3,501 | .617  .661  .613  .603  .791  .667  .631 | .523  .439  .473  .499  .441  .562  .452 | .728  .996  .795  .728  1.421  .791  .881 | *F*(1, 19) = 0.107, *p* = .747  *R^2^* = .000  *F*(1, 18) = 0.013, *p* = .912  *R^2^* = .000  *F*(2, 15) = 0.256, *p* = .777  *R^2^* = .000 |
| *Lower extremity injuries* | | | | | | |
| Design  Randomised  Non-randomised  Equipment  With  Without  Monitoring  Researcher  Trainer  Players | 14  3  5  12  1  11  2 | 13,339  559  4,439  9,459  194  10,989  1,664 | .628  .657  .805  .582  .817  .638  .775 | .524  .435  .622  .492  .452  .526  .510 | .752  0.992  1.044  .689  1.474  .775  1.179 | *F*(1, 15) = 0.047, *p* = .831  *R^2^* = .000  *F*(1, 15) = 4.999, *p* = .041  *R^2^* = .351  *F*(2, 11) = 0.713, *p* = .512  *R^2^* = .000 |
| *Thigh injuries* | | | | | | |
| Design  Randomised  Non-randomised  Equipment  With  Without  Monitoring  Researcher  Trainer | 11  2  2  11  1  9 | 11,157  268  2,257  9,168  194  10,180 | .707  .678  1.237  .616  .713  .699 | .487  .322  .768  .468  .217  .420 | 1.025  1.425  1.991  .811  2.345  1.162 | *F*(1, 11) = 0.012, *p* = .914  *R^2^* = .000  *F*(1, 11) = 7.787, *p* = .018  *R^2^* = 1.000  *F*(1, 8) = 0.001, *p* = .973  *R^2^* = .000 |
| *Knee injuries* | | | | | | |
| Design  Randomised  Non-randomised  Equipment  With  Without  Monitoring  Researcher  Trainer | 11  2  2  11  1  9 | 11,157  268  2,257  9,168  194  10,180 | .628  .972  .997  .617  .697  .663 | .468  .421  .497  .463  .238  .479 | .845  2.246  2.000  .822  2.039  .919 | *F*(1, 11) = 1.171, *p* = .302  *R^2^* = .046  *F*(1, 11) = 1.970, *p* = .188  *R^2^* = 0.239  *F*(1, 8) = 0.011, *p* = .921  *R^2^* = .000 |
| *Ankle injuries* | | | | | | |
| Design  Randomised  Non-randomised  Equipment  With  Without  Monitoring  Researcher  Trainer | 11  2  2  11  1  9 | 11,157  268  2,257  9,168  194  10,180 | .614  .548  .690  .588  1.141  .697 | .414  .201  .295  .392  .419  .499 | .911  1.497  1.613  .881  3.106  0.974 | *F*(1, 11) = 0.053, *p* = .822  *R^2^* = .000  *F*(1, 11) = 0.140, *p* = .715  *R^2^* = .000  *F*(1, 8) = 1.155, *p* = .314  *R^2^* = .039 |

*Note. k* = number of independent samples for each category of the qualitative moderator variable; N = total sample size for each category of the qualitative moderator variable; ${IRR}_{+}$= pooled effect size estimate (Incidence rate ratio) for each category of the qualitative moderator variable; LL = lower limit of the 95% confidence interval; UL = upper limit of the 95% confidence interval; *F* ()= Knapp-Hartung’s statistic for testing the significance of the moderator variable and its degrees of freedom; *p* = *p*-value for the Knapp-Hartung’s *F* statistic; *R^2^* = proportion of variance accounted for by the moderator.
