# Supplementary material for: A systematic review and network meta-analysis on the effectiveness of exercise-based interventions for reducing the injury incidence in youth team-sport players. Part 1: an analysis by classical training components
Source: Ann Med. 2024 Oct 1;56(1):2408457. doi: 10.1080/07853890.2024.2408457 (PMC11445890; doi:10.1080/07853890.2024.2408457)
Supplement: Supplemental Material [file IANN_A_2408457_SM0607.zip › suppl_data/Supplementary file 13.docx]

| **Supplementary file 13.** Results of the simple meta-regressions applied on the overall, lower extremity, thigh, knee, and ankle injuries estimates, taking continuous moderator variables as predictors. | | | | | | | | |
| --- | --- | --- | --- | --- | --- | --- | --- | --- |
| **Predictor** | ***k*** | **N** | $\boldsymbol{b}_{\boldsymbol{j}}$ | **LL** | **UL** | ***F* (df)** | ***p*** | $\mathbf{R}^{\mathbf{2}}$ |
| *Overall injuries* | | | | | | | | |
| Duration | 20 | 18,111 | .004 | -.012 | .019 | 0.254 (1,18) | .620 | .000 |
| Weeks | 21 | 18,305 | .000 | -.015 | .015 | 0.000 (1,19) | .996 | .000 |
| Male rate | 20 | 14,410 | .117 | -.291 | .525 | 0.363 (1,18) | .554 | .000 |
| *Lower extremity* | | | | | | | | |
| Duration | 16 | 13,704 | -.013 | -.037 | .012 | 1.232 (1,14) | .286 | .027 |
| Weeks | 17 | 13,898 | -.001 | -.018 | .015 | 0.022 (1,15) | .883 | .000 |
| Male rate | 16 | 10,003 | .421 | .046 | .796 | 5.800 (1,14) | .030 | .411 |
| *Thigh injuries* | | | | | | | | |
| Duration | 12 | 11,231 | .010 | -.080 | .101 | 0.066 (1,10) | .802 | .000 |
| Weeks | 13 | 11,425 | -.011 | -.044 | .022 | 0.548 (1,11) | .475 | .000 |
| Male rate | 12 | 7,530 | .666 | .058 | 1.274 | 5.962 (1,10) | .035 | 1.000 |
| *Knee injuries* | | | | | | | | |
| Duration | 12 | 11,231 | 0.05 | -0.034 | 0.133 | 1.737 (1,10) | .217 | .178 |
| Weeks | 13 | 11,425 | -0.002 | -0.036 | 0.031 | 0.024 (1,11) | .879 | .000 |
| Male rate | 12 | 7,530 | 0.222 | -0.55 | 0.995 | 0.411 (1,10) | .536 | .000 |
| *Ankle injuries* | | | | | | | | |
| Duration | 12 | 11,231 | -0.079 | -0.157 | -0.001 | 5.102 (1,10) | .047 | .504 |
| Weeks | 13 | 11,425 | -0.009 | -0.046 | 0.028 | 0.276 (1,11) | .610 | .000 |
| Male rate | 12 | 7,530 | 0.358 | -0.523 | 1.238 | 0.820 (1,10) | .387 | .000 |

*Note. k* = number of independent samples included in the analysis; N = total sample size; $b_{j}$ = regression coefficient of each predictor; LL = lower limit of the 95% confidence interval for the regression coefficient; UL = upper limit of the 95% confidence interval for the regression coefficient; *F* (df) = Knapp-Hartung’s statistic for testing the significance of the moderator variable and its degrees of freedom; *p* = *p*-value for the Knapp-Hartung’s *F* statistic; *R^2^* = proportion of variance accounted for by the moderator.
