# Supplementary material for: A systematic review and network meta-analysis on the effectiveness of exercise-based interventions for reducing the injury incidence in youth team-sport players. Part 1: an analysis by classical training components
Source: Ann Med. 2024 Oct 1;56(1):2408457. doi: 10.1080/07853890.2024.2408457 (PMC11445890; doi:10.1080/07853890.2024.2408457)
Supplement: Supplemental Material [file IANN_A_2408457_SM0607.zip › suppl_data/Supplementary file 14.pdf]

Supplementary file 14. Funnel plots and results of the Egger's regression test for detecting the threat of publication bias for the meta-analyses conducted for overall, lower extremity, thigh, knee, and ankle injuries.

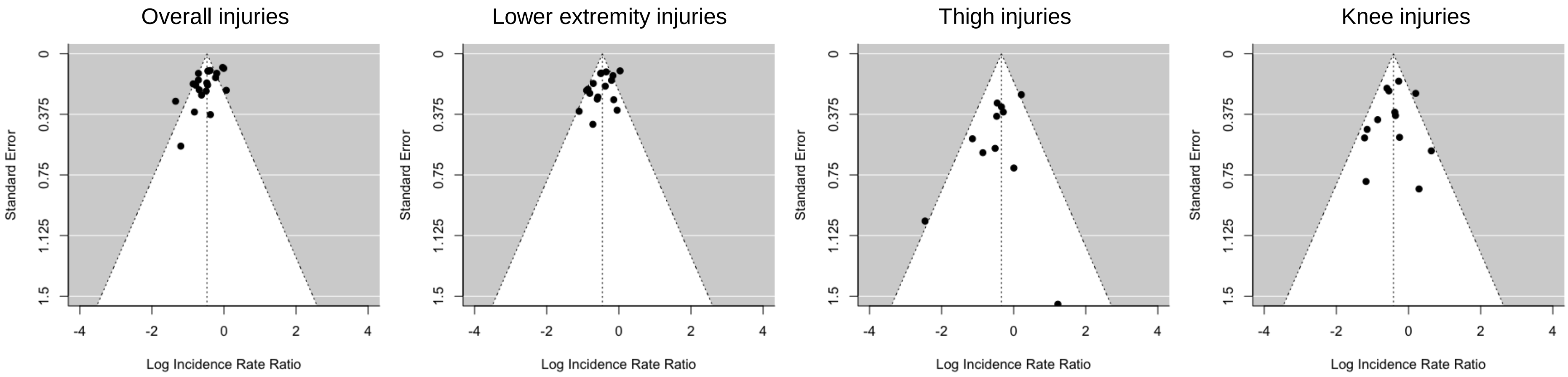

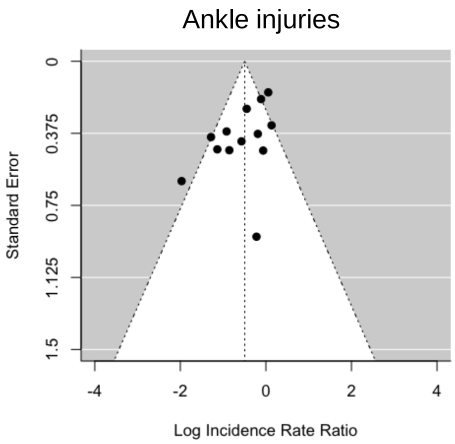

Injuries	*b*	SE_b	t	*df*	*p*
Overall	-2.729	0.885	-3.08	19	0.006
Lower extremity	-2.027	0.938	-2.16	15	0.047
Thigh	-0.515	0.620	-0.83	11	0.424
Knee	-0.444	0.810	-0.55	11	0.594
Ankle	-2.315	0.819	-2.82	11	0.017

Note. b = Egger's regression coefficient estimate; SE_b = standard error for the Egger's regression coefficient estimate; t = statistic that follows a Student's T distribution with *k*-2 degrees of freedom; *df* = degrees of freedom for the t statistic; *p* = *p*-value for the t statistic.
