# Supplementary material for: A systematic review and network meta-analysis on the effectiveness of exercise-based interventions for reducing the injury incidence in youth team-sport players. Part 1: an analysis by classical training components
Source: Ann Med. 2024 Oct 1;56(1):2408457. doi: 10.1080/07853890.2024.2408457 (PMC11445890; doi:10.1080/07853890.2024.2408457)
Supplement: Supplemental Material [file IANN_A_2408457_SM0607.zip › suppl_data/Supplementary file 15.docx]

**Supplementary file 15a.** Results of the network meta-analysis under the comprehensive approach for lower extremity injuries: Estimates and 95% confidence intervals for comparisons between each pair of programs (programs in rows vs. programs in columns).

| **Program** | | | | | | | | | | |
| --- | --- | --- | --- | --- | --- | --- | --- | --- | --- | --- |
| 1 | 1 |  |  |  |  |  |  |  |  |  |
| 3 | 0.549  [0.317, 0.952] | 3 |  |  |  |  |  |  |  |  |
| 5 | 0.493  [0.343, 0.707] | 0.897  [0.464, 1.732] | 5 |  |  |  |  |  |  |  |
| 7 | 0.624  [0.515, 0.757] | 1.137  [0.635, 2.037] | 1.268  [0.842, 1.908] | 7 |  |  |  |  |  |  |
| 8 | 0.583  [0.505, 0.673] | 1.061  [0.601, 1.875] | 1.183  [0.802, 1.745] | 0.933  [0.734, 1.187] | 8 |  |  |  |  |  |
| 9 | 1.035  [0.841, 1.275] | 1.885  [1.046, 3.395] | 2.101  [1.385, 3.188] | 1.658  [1.248, 2.202] | 1.776  [1.379, 2.288] | 9 |  |  |  |  |
| 10 | 0.763  [0.591, 0.985] | 1.389  [0.757, 2.548] | 1.549  [0.995, 2.41] | 1.222  [0.887, 1.683] | 1.309  [0.976, 1.755] | 0.737  [0.53, 1.025] | 10 |  |  |  |
| 11 | 0.848  [0.65, 1.106] | 1.544  [0.838, 2.845] | 1.721  [1.099, 2.695] | 1.358  [0.978, 1.886] | 1.455  [1.075, 1.969] | 0.819  [0.584, 1.148] | 1.111  [0.768, 1.607] | 11 |  |  |
| 12 | 0.333  [0.165, 0.669] | 0.606  [0.249, 1.474] | 0.675  [0.308, 1.483] | 0.533  [0.258, 1.1] | 0.571  [0.28, 1.165] | 0.321  [0.155, 0.666] | 0.436  [0.207, 0.917] | 0.392  [0.186, 0.829] | 12 |  |
| 13 | 0.687  [0.463, 1.019] | 1.25  [0.635, 2.461] | 1.394  [0.817, 2.38] | 1.1  [0.709, 1.706] | 1.178  [0.774, 1.793] | 0.663  [0.425, 1.036] | 0.9  [0.563, 1.44] | 0.81  [0.503, 1.303] | 2.064  [0.926, 4.604] | 13 |

*Note*. Program 1 = Control; Program 3 = Stability; Program 5 = Flexibility; Program 7 = Strength + plyometric + stability + speed & agility + warm-up drills; Program 8 = Strength + plyometric + stability; Program 9 = Strength + plyometric + stability + speed & agility; Program 10 = Strength + plyometric + stability + speed & agility + warm-up drills + flexibility; Program 11 = Stability + flexibility; Program 12 = Strength + stability; Program 13 = Strength + plyometric + stability + warm-up drills + flexibility. Values below 1 favor the row intervention (IPP).

**Supplementary file 15b.** Results of the network meta-analysis under the comprehensive approach for thigh injuries: Estimates and 95% confidence intervals for comparisons between each pair of programs (programs in rows vs. programs in columns).

| **Program** | | | | | | | | |
| --- | --- | --- | --- | --- | --- | --- | --- | --- |
| 1 | 1 |  |  |  |  |  |  |  |
| 3 | 0.598  [0.19, 1.884] | 3 |  |  |  |  |  |  |
| 5 | 0.318  [0.114, 0.893] | 0.533  [0.114, 2.492] | 5 |  |  |  |  |  |
| 7 | 0.618  [0.321, 1.188] | 1.033  [0.276, 3.871] | 1.939  [0.572, 6.577] | 7 |  |  |  |  |
| 8 | 0.647  [0.428, 0.978] | 1.082  [0.319, 3.663] | 2.03  [0.668, 6.168] | 1.047  [0.483, 2.269] | 8 |  |  |  |
| 9 | 1.237  [0.752, 2.033] | 2.069  [0.592, 7.226] | 3.884  [1.236, 12.203] | 2.003  [0.881, 4.552] | 1.913  [1.002, 3.651] | 9 |  |  |
| 10 | 0.713  [0.374, 1.359] | 1.193  [0.32, 4.449] | 2.239  [0.663, 7.557] | 1.154  [0.461, 2.892] | 1.103  [0.512, 2.373] | 0.576  [0.255, 1.301] | 10 |  |
| 12 | 1.231  [0.024, 62.037] | 2.059  [0.035, 122.335] | 3.865  [0.067, 222.597] | 1.993  [0.037, 106.028] | 1.904  [0.037, 98.054] | 0.995  [0.019, 51.755] | 1.727  [0.032, 91.724] | 12 |

*Note*. Program 1 = Control; Program 3 = Stability; Program 5 = Flexibility; Program 7 = Strength + plyometric + stability + speed & agility + warm-up drills; Program 8 = Strength + plyometric + stability; Program 9 = Strength + plyometric + stability + speed & agility; Program 10 = Strength + plyometric + stability + speed & agility + warm-up drills + flexibility; Program 12 = Strength + stability. Values below 1 favor the row intervention (IPP).

**Supplementary file 15c.** Results of the network meta-analysis under the comprehensive approach for knee injuries: Estimates and 95% confidence intervals for comparisons between each pair of programs (programs in rows vs. programs in columns).

| **Program** | | | | | | | | |
| --- | --- | --- | --- | --- | --- | --- | --- | --- |
| 1 | 1 |  |  |  |  |  |  |  |
| 3 | 1.883  [0.58, 6.115] | 3 |  |  |  |  |  |  |
| 5 | 0.296  [0.107, 0.821] | 0.157  [0.033, 0.746] | 5 |  |  |  |  |  |
| 7 | 0.604  [0.425, 0.86] | 0.321  [0.094, 1.097] | 2.043  [0.694, 6.019] | 7 |  |  |  |  |
| 8 | 0.633  [0.499, 0.803] | 0.336  [0.101, 1.118] | 2.14  [0.75, 6.107] | 1.047  [0.684, 1.603] | 8 |  |  |  |
| 9 | 1.22  [0.754, 1.975] | 0.648  [0.182, 2.313] | 4.127  [1.334, 12.762] | 2.02  [1.112, 3.668] | 1.928  [1.127, 3.3] | 9 |  |  |
| 10 | 0.697  [0.329, 1.476] | 0.37  [0.092, 1.496] | 2.357  [0.664, 8.369] | 1.154  [0.504, 2.643] | 1.101  [0.501, 2.42] | 0.571  [0.234, 1.393] | 10 |  |
| 12 | 0.308  [0.065, 1.449] | 0.163  [0.023, 1.144] | 1.041  [0.163, 6.656] | 0.509  [0.104, 2.495] | 0.486  [0.101, 2.332] | 0.252  [0.05, 1.278] | 0.441  [0.079, 2.469] | 12 |

*Note*. Program 1 = Control; Program 3 = Stability; Program 5 = Flexibility; Program 7 = Strength + plyometric + stability + speed & agility + warm-up drills; Program 8 = Strength + plyometric + stability; Program 9 = Strength + plyometric + stability + speed & agility; Program 10 = Strength + plyometric + stability + speed & agility + warm-up drills + flexibility; Program 12 = Strength + stability. Values below 1 favor the row intervention (IPP).

**Supplementary file 15d.** Results of the network meta-analysis under the comprehensive approach for ankle injuries: Estimates and 95% confidence intervals for comparisons between each pair of programs (programs in rows vs. programs in columns).

| **Program** | | | | | | | | |
| --- | --- | --- | --- | --- | --- | --- | --- | --- |
| 1 | 1 |  |  |  |  |  |  |  |
| 3 | 0.139  [0.041, 0.474] | 3 |  |  |  |  |  |  |
| 5 | 0.828  [0.395, 1.737] | 5.936  [1.422, 24.787] | 5 |  |  |  |  |  |
| 7 | 0.746  [0.535, 1.042] | 5.351  [1.507, 18.999] | 0.901  [0.4, 2.032] | 7 |  |  |  |  |
| 8 | 0.542  [0.394, 0.746] | 3.888  [1.099, 13.75] | 0.655  [0.292, 1.467] | 0.727  [0.458, 1.153] | 8 |  |  |  |
| 9 | 1.056  [0.769, 1.45] | 7.573  [2.142, 26.771] | 1.276  [0.57, 2.856] | 1.415  [0.893, 2.243] | 1.948  [1.243, 3.053] | 9 |  |  |
| 10 | 1.141  [0.594, 2.192] | 8.178  [2.045, 32.7] | 1.378  [0.513, 3.699] | 1.528  [0.734, 3.183] | 2.103  [1.017, 4.351] | 1.08  [0.522, 2.232] | 10 |  |
| 12 | 0.321  [0.131, 0.789] | 2.302  [0.505, 10.494] | 0.388  [0.121, 1.243] | 0.43  [0.165, 1.122] | 0.592  [0.228, 1.536] | 0.304  [0.117, 0.788] | 0.282  [0.093, 0.855] | 12 |

*Note*. Program 1 = Control; Program 3 = Stability; Program 5 = Flexibility; Program 7 = Strength + plyometric + stability + speed & agility + warm-up drills; Program 8 = Strength + plyometric + stability; Program 9 = Strength + plyometric + stability + speed & agility; Program 10 = Strength + plyometric + stability + speed & agility + warm-up drills + flexibility; Program 12 = Strength + stability. Values below 1 favor the row intervention (IPP).
