# Supplementary material for: A systematic review and network meta-analysis on the effectiveness of exercise-based interventions for reducing the injury incidence in youth team-sport players. Part 1: an analysis by classical training components
Source: Ann Med. 2024 Oct 1;56(1):2408457. doi: 10.1080/07853890.2024.2408457 (PMC11445890; doi:10.1080/07853890.2024.2408457)
Supplement: Supplemental Material [file IANN_A_2408457_SM0607.zip › suppl_data/Supplementary file 16.docx]

**Supplementary file 16a.** Results of the network meta-analysis under the restrictive approach for lower extremity injuries: Estimates and 95% confidence intervals for comparisons between each pair of programs (programs in rows vs. programs in columns).

| **Program** | | | | | | | | | | |
| --- | --- | --- | --- | --- | --- | --- | --- | --- | --- | --- |
| 1 | 1 |  |  |  |  |  |  |  |  |  |
| 3 | 0.848  [0.65, 1.106] | 3 |  |  |  |  |  |  |  |  |
| 5 | 0.493  [0.343, 0.707] | 0.581  [0.371, 0.91] | 5 |  |  |  |  |  |  |  |
| 6 | 0.868  [0.497, 1.518] | 1.024  [0.552, 1.901] | 1.763  [0.907, 3.427] | 6 |  |  |  |  |  |  |
| 7 | 0.624  [0.515, 0.757] | 0.736  [0.53, 1.023] | 1.268  [0.842, 1.908] | 0.719  [0.398, 1.298] | 7 |  |  |  |  |  |
| 8 | 0.537  [0.443, 0.651] | 0.633  [0.456, 0.879] | 1.09  [0.724, 1.641] | 0.618  [0.343, 1.116] | 0.86  [0.655, 1.129] | 8 |  |  |  |  |
| 10 | 0.953  [0.48, 1.892] | 1.124  [0.539, 2.345] | 1.935  [0.892, 4.198] | 1.098  [0.454, 2.657] | 1.527  [0.749, 3.111] | 1.776  [0.872, 3.618] | 10 |  |  |  |
| 14 | 0.614  [0.485, 0.777] | 0.724  [0.508, 1.033] | 1.247  [0.81, 1.918] | 0.707  [0.386, 1.296] | 0.984  [0.726, 1.333] | 1.144  [0.844, 1.55] | 0.644  [0.312, 1.329] | 14 |  |  |
| 15 | 0.687  [0.463, 1.019] | 0.81  [0.503, 1.303] | 1.394  [0.817, 2.38] | 0.791  [0.399, 1.567] | 1.1  [0.709, 1.706] | 1.279  [0.825, 1.984] | 0.72  [0.327, 1.588] | 1.118  [0.707, 1.77] | 15 |  |
| 17 | 0.559  [0.331, 0.946] | 0.66  [0.366, 1.189] | 1.136  [0.601, 2.148] | 0.644  [0.299, 1.387] | 0.896  [0.512, 1.567] | 1.042  [0.596, 1.823] | 0.587  [0.248, 1.391] | 0.911  [0.512, 1.619] | 0.815  [0.422, 1.571] | 17 |

*Note.* Program 1 = Control; Program 3 = Stability; Program 5 = Flexibility; Program 6 = Plyometrics + stability; Program 7 = Strength + plyometrics + stability + speed & agility + drills; Program 8 = Strength + plyometrics + stability; Program 10 = Strength + plyometrics + stability + speed & agility + drills + flexibility; Program 14 = Strength + plyometrics; Program 15 = Stability + drills + flexibility; Program 17 = Plyometrics + stability + speed & agility + drills + flexibility. Values below 1 favor the row intervention.

**Supplementary file 16b.** Results of the network meta-analysis under the restrictive approach for thigh injuries: Estimates and 95% confidence intervals for comparisons between each pair of programs (programs in rows vs. programs in columns).

| **Program** | | | | | | |
| --- | --- | --- | --- | --- | --- | --- |
| 1 | 1 |  |  |  |  |  |
| 5 | 0.318  [0.114, 0.893] | 5 |  |  |  |  |
| 6 | 3.411  [0.164, 71.057] | 10.712  [0.434, 264.568] | 6 |  |  |  |
| 7 | 0.618  [0.321, 1.188] | 1.939  [0.572, 6.577] | 0.181  [0.008, 4.043] | 7 |  |  |
| 8 | 0.628  [0.381, 1.035] | 1.971  [0.627, 6.2] | 0.184  [0.008, 3.992] | 1.016  [0.446, 2.314] | 8 |  |
| 14 | 0.624  [0.292, 1.333] | 1.96  [0.544, 7.052] | 0.183  [0.008, 4.184] | 1.01  [0.371, 2.752] | 0.994  [0.401, 2.467] | 14 |

*Note.* Program 1 = Control; Program 5 = Flexibility; Program 6 = Plyometrics + stability; Program 7 = Strength + plyometrics + stability + speed & agility + drills; Program 8 = Strength + plyometrics + stability; Program 14 = Strength + plyometrics. Values below 1 favor the row intervention.

**Supplementary file 16c.** Results of the network meta-analysis under the restrictive approach for knee injuries: Estimates and 95% confidence intervals for comparisons between each pair of programs (programs in rows vs. programs in columns).

| **Program** | | | | | | |
| --- | --- | --- | --- | --- | --- | --- |
| 1 | 1 |  |  |  |  |  |
| 5 | 0.296  [0.107, 0.821] | 5 |  |  |  |  |
| 6 | 0.78  [0.283, 2.15] | 2.637  [0.625, 11.121] | 6 |  |  |  |
| 7 | 0.604  [0.425, 0.86] | 2.043  [0.694, 6.019] | 0.775  [0.265, 2.268] | 7 |  |  |
| 8 | 0.496  [0.346, 0.712] | 1.677  [0.568, 4.955] | 0.636  [0.217, 1.867] | 0.821  [0.495, 1.36] | 8 |  |
| 14 | 0.762  [0.546, 1.064] | 2.577  [0.88, 7.545] | 0.977  [0.336, 2.843] | 1.261  [0.776, 2.05] | 1.536  [0.939, 2.513] | 14 |

*Note.* Program 1 = Control; Program 5 = Flexibility; Program 6 = Plyometrics + stability; Program 7 = Strength + plyometrics + stability + speed & agility + drills; Program 8 = Strength + plyometrics + stability; Program 14 = Strength + plyometrics. Values below 1 favor the row intervention.

**Supplementary file 16d.** Results of the network meta-analysis under the restrictive approach for ankle injuries: Estimates and 95% confidence intervals for comparisons between each pair of programs (programs in rows vs. programs in columns).

| **Program** | | | | | | |
| --- | --- | --- | --- | --- | --- | --- |
| 1 | 1 |  |  |  |  |  |
| 5 | 0.828  [0.395, 1.737] | 5 |  |  |  |  |
| 6 | 0.938  [0.377, 2.332] | 1.133  [0.35, 3.665] | 6 |  |  |  |
| 7 | 0.746  [0.535, 1.042] | 0.901  [0.4, 2.032] | 0.796  [0.302, 2.099] | 7 |  |  |
| 8 | 0.579  [0.397, 0.846] | 0.7  [0.305, 1.608] | 0.618  [0.23, 1.656] | 0.776  [0.469, 1.286] | 8 |  |
| 14 | 0.277  [0.128, 0.601] | 0.335  [0.115, 0.977] | 0.296  [0.09, 0.976] | 0.372  [0.16, 0.863] | 0.479  [0.202, 1.132] | 14 |

*Note.* Program 1 = Control; Program 5 = Flexibility; Program 6 = Plyometrics + stability; Program 7 = Strength + plyometrics + stability + speed & agility + drills; Program 8 = Strength + plyometrics + stability; Program 14 = Strength + plyometrics. Values below 1 favor the row intervention.
