# Supplementary material for: A systematic review and network meta-analysis on the effectiveness of exercise-based interventions for reducing the injury incidence in youth team-sport players. Part 1: an analysis by classical training components
Source: Ann Med. 2024 Oct 1;56(1):2408457. doi: 10.1080/07853890.2024.2408457 (PMC11445890; doi:10.1080/07853890.2024.2408457)
Supplement: Supplemental Material [file IANN_A_2408457_SM0607.zip › suppl_data/Supplementary file 17.docx]

| **Supplementary file 17.** Results of the simple meta-regressions applied on the overall, lower extremity, thigh, knee, and ankle injuries estimates, taking CONSORT, PEDro, and Risk of Bias scales as predictors. | | | | | | | | |
| --- | --- | --- | --- | --- | --- | --- | --- | --- |
| **Predictor** | ***k*** | **N** | $\boldsymbol{b}_{\boldsymbol{j}}$ | **LL** | **UL** | ***F* (df)** | ***p*** | $\mathbf{R}^{\mathbf{2}}$ |
| *Overall injuries* | | | | | | | | |
| CONSORT | 21 | 18,305 | -.005 | -.033 | .023 | 0.159 (1,19) | .695 | 0.00 |
| PEDro | 21 | 18,305 | -.020 | -.089 | .049 | 0.363 (1,19) | .554 | 0.00 |
| Risk of bias | 21 | 18,305 | -.005 | -.093 | .082 | 0.017 (1,19) | .898 | 0.00 |
| *Lower extremity* | | | | | | | | |
| CONSORT | 17 | 13,898 | -0.001 | -.030 | .029 | 0.002 (1,15) | .968 | .000 |
| PEDro | 17 | 13,898 | -0.007 | -.085 | .070 | 0.042 (1,15) | .840 | .000 |
| Risk of bias | 17 | 13,898 | 0.029 | -.065 | .123 | 0.419 (1,15) | .527 | .000 |
| *Thigh injuries* | | | | | | | | |
| CONSORT | 13 | 11,425 | 0.007 | -.053 | .067 | 0.062 (1,11) | .808 | .000 |
| PEDro | 13 | 11,425 | -0.018 | -.175 | .138 | 0.066 (1,11) | .802 | .000 |
| Risk of bias | 13 | 11,425 | 0.009 | -.175 | .194 | 0.012 (1,11) | .915 | .000 |
| *Knee injuries* | | | | | | | | |
| CONSORT | 13 | 11,425 | 0.023 | -.026 | .072 | 1.048 (1,11) | .328 | .013 |
| PEDro | 13 | 11,425 | 0.051 | -.105 | .206 | 0.512 (1,11) | .489 | .000 |
| Risk of bias | 13 | 11,425 | 0.093 | -.077 | .263 | 1.449 (1,11) | .254 | 0.117 |
| *Ankle injuries* | | | | | | | | |
| CONSORT | 13 | 11,425 | -0.013 | -.071 | .044 | 0.267 (1,11) | .616 | .000 |
| PEDro | 13 | 11,425 | -0.042 | -.221 | .136 | 0.276 (1,11) | .610 | .000 |
| Risk of bias | 13 | 11,425 | -0.026 | -.242 | .189 | 0.072 (1,11) | .793 | .000 |

*Note. k* = number of independent samples included in the analysis; N = total sample size; $b_{j}$ = regression coefficient of each predictor; LL = lower limit of the 95% confidence interval for the regression coefficient; UL = upper limit of the 95% confidence interval for the regression coefficient; *F* (df) = Knapp-Hartung’s statistic for testing the significance of the moderator variable and its degrees of freedom; *p* = *p*-value for the Knapp-Hartung’s *F* statistic; *R^2^* = proportion of variance accounted for by the moderator.
