# Supplementary material for: A systematic review and network meta-analysis on the effectiveness of exercise-based interventions for reducing the injury incidence in youth team-sport players. Part 1: an analysis by classical training components
Source: Ann Med. 2024 Oct 1;56(1):2408457. doi: 10.1080/07853890.2024.2408457 (PMC11445890; doi:10.1080/07853890.2024.2408457)
Supplement: Supplemental Material [file IANN_A_2408457_SM0607.zip › suppl_data/Supplementary file 2.docx]

| **Supplementary file 2**. Inclusion/exclusion criteria for young team-sport players' injuries literature search. | | | |
| --- | --- | --- | --- |
|  | **Inclusion criteria** | **Exclusion criteria** | **Rationale for these criteria** |
| Publication type | Peer-reviewed original research articles only. | Non-peer-reviewed articles, newspapers, opinion pieces, systematic reviews and meta-analyses, editorials, commentaries, and letters to the editor.  Conference proceedings/abstracts.  Book chapters. | For reasons of practicality and to avoid duplication of data, it was deemed acceptable to include only studies published in peer-reviewed journals. |
| Language | English and Spanish | Non- English and Spanish. | According to the researchers’ proficiency, it was deemed acceptable to include only studies published in English or Spanish. |
| Publication date | Up to 15^th^ January 2024. | - | All articles were included regardless of the time period. |
| Study design | Randomised and non-randomised controlled trials. | Any study design that did not compare the intervention with a control group, or with another intervention recognized as a common comparator (i.e., a different node) in the network diagram. | Every study that meets the rest of the eligibility criteria and compares any pair of the eligible interventions (e.g., intervention vs. control; intervention 1 vs. intervention 2) was included in this NMA. The only requirement was that one of the interventions implemented in the original study involved exactly one common comparator (e.g., control group), so that they were “connected”. |
| Type of intervention | Exercise-based strategies comprised of one or multiple exercise components (e.g., strength, plyometrics, flexibility, stability) implemented as warm-ups’ protocols and/or independent training interventions with the aim of reducing injury incidence. | Non-exercise-based interventions (e.g., protective equipment) and/or those exercise-based strategies using a co-intervention (e.g., education). | The purpose was to analyse exercise-based interventions for reducing the injury risk, so those interventions aimed to reduce injuries but focused on other aspects different to physical exercise (e.g., protective equipment) were excluded. Studies implementing both physical exercise and a non-exercise co-intervention that was not applied in all the intervention groups were excluded. |
| Sex and age | Male and female team sport players younger than or equal to 19 years old. | Studies with no team sport players, where the players were older than 19 years old or participants’ age was not specified. | The primary outcome of interest was the efficacy of exercise interventions for reducing the injury incidence in youth team-sport players, so only those studies in male and female players younger than 19 years old were included. Studies were included irrespective of whether they analysed pooled or separate data for both sexes. |
| Playing level | Participating in elite and/or sub-elite/amateur level. | Non-competitive sport activities. | Variations in injury definitions and data collection procedures probably applied in non-competitive environments, as well as the absence of competition (where, in fact, most injuries occur), might affect the reported efficacy of exercise interventions in youth. Therefore, only studies analysing the effect of these interventions in young players participating in games/training in a competitive team-sport setting (elite and sub-elite) were included. Elite involved professional youth academies, national teams and international tournaments. Players not described as belonging to a professional club academy, playing at a high level or classified as elite were considered as sub-elite (e.g., community, regional and inter-provincial playing levels). |
| Injury definition | Time-loss and medical attention injury definitions mentioned previously in the FIFA (Fuller et al., 2006) and later in the IOC (Bahr et al. 2020) Consensus Statements were followed. Studies had to report overall injury incidents. | Other injury definitions (e.g., at least 48 h of absence from sport post incident). Studies that only reported specific injury types and/or locations (e.g., ACL, knee injuries, etc.). | Injuries that result in an athlete receiving medical attention (“medical attention” definition) and injuries that result in a player being unable to complete the current or future training session or competition (“time-loss” definition) were considered. Studies with other definitions would not meet the methodological criteria of the meta-analysis, as they might increase inconsistencies between data collection procedures. |
