# Supplementary material for: A systematic review and network meta-analysis on the effectiveness of exercise-based interventions for reducing the injury incidence in youth team-sport players. Part 1: an analysis by classical training components
Source: Ann Med. 2024 Oct 1;56(1):2408457. doi: 10.1080/07853890.2024.2408457 (PMC11445890; doi:10.1080/07853890.2024.2408457)
Supplement: Supplemental Material [file IANN_A_2408457_SM0607.zip › suppl_data/Supplementary file 3.docx]

**Supplementary file 3.** Search strategy

**Search strategy in PubMed** **- 1923 results**

#1 (injury prevention[tiab] OR intervention[tiab] OR program*[tiab] OR warm-up[tiab] OR 11+[tiab] OR neuromuscular[tiab]) AND (injur*[tiab]) AND (children[tiab] OR adolescent[tiab] OR youth[tiab]) AND (team[tiab] OR sport[tiab] OR soccer[tiab] OR football[tiab] OR rugby[tiab] OR basketball[tiab] OR volleyball[tiab] OR handball[tiab] OR hockey[tiab])

#2 #1 Filters: Published up to 15^th^ January 2024.

**Search strategy in Web of Science - 200 results**

#1 TITLE: (prevent* OR intervention OR program* OR warm-up OR 11+ OR neuromuscular) AND TITLE: (injur*) AND TITLE: (child* OR adolescent* OR youth) AND TITLE: (team OR sport OR soccer OR football OR rugby OR basketball OR volleyball OR handball OR hockey)

#2 Filters: Published up to 15^th^ January 2024.

**Search strategy in the Cochrane Central Register of Controlled Trials - 1133 results**

#1 (prevent* OR intervention OR program* OR warm-up OR 11 OR neuromuscular) [Title/Abstract/Key Word] AND (injur*) [Title/Abstract/Key Word] AND (child* OR adolescent* OR youth) [Title/Abstract/Key Word] (team OR sport OR soccer OR football OR rugby OR basketball OR volleyball OR handball OR hockey) [Title/Abstract/Key Word]

#2 Filters: Trials

#3 Filters: Published up to 15^th^ January 2024.

**Search strategy in SPORTDiscus - 1358 results**

#1 AB (prevent* or intervention or program* or warm-up or 11+ or neuromuscular) AND AB (injur*) AND AB (child* or adolescent* or youth) AND AB (team or sport or soccer or football or rugby or basketball or volleyball or handball or hockey)

#2 Filters: Published up to 15^th^ January 2024.
