# Supplementary material for: A systematic review and network meta-analysis on the effectiveness of exercise-based interventions for reducing the injury incidence in youth team-sport players. Part 1: an analysis by classical training components
Source: Ann Med. 2024 Oct 1;56(1):2408457. doi: 10.1080/07853890.2024.2408457 (PMC11445890; doi:10.1080/07853890.2024.2408457)
Supplement: Supplemental Material [file IANN_A_2408457_SM0607.zip › suppl_data/Supplementary file 4.docx]

| **Supplementary file 4.** Moderator variables coded. |
| --- |
| **General study descriptors** |
| - Authors. - Year of the study. - Country. - Study design (randomized control trial, non-randomized control trial). - Reporting quality (CONSORT scale). - Methodological quality (PEDro scale). - Risk of bias (Cochrane Back and Neck Group scale). |
| **Description of the study population** |
| - Sample size (*n*). - Team sport. - Number of teams. - Group (intervention or control group). - Level of play (amateur or elite). - Sex (male, female or mixed). - Age (mean ± SD and/or 95%CI). |
| **Characteristics of the intervention** |
| - Length (weeks). - Weekly frequency (days). - Duration. The time (minutes) spent for completing the IPP in each session. - Volume. The total number of training sessions in which IPP was delivered throughout the length of the intervention period. - IPP modality (S-IPP or M-IPP). - Equipment required (no or yes [indicating the type]). - Who delivered the intervention (researcher, coach, or player). - Type/s of exercise component/s integrated into the IPP:   - Strength. Eccentric, concentric and/or isometric strength training (e.g., Nordic hamstring, squats), weight training, core strength (e.g., sit-ups).   - Speed/Agility. Running exercises at maximum speed, integration of COD actions, and sports-specific drills (e.g., linear sprint, planned plant and cut).   - Plyometric. Jumping and rebounding exercises (e.g., skating jumps, bounding).   - Stability. Proprioception, neuromuscular training with wobble boards or balance mats, core stability (e.g., planks), body control or one-leg coordination (e.g., skater figure and cross-country skiing exercises).   - Coordination/warm-up drills. Basic (e.g., skipping, heel flicks, hip out/in, carioca) and specific (e.g., defensive pressure technique) warm-up drills.   - Flexibility. Dynamic and/or static stretching exercises. |
| **Epidemiological descriptors** |
| - Injury definition. - Number of injuries (overall, lower extremity, thigh, knee and/or ankle). - Sport exposure (total hours of players exposure). - Injury incidence (injuries per 1000h of sport exposure). |
