# Supplementary material for: A systematic review and network meta-analysis on the effectiveness of exercise-based interventions for reducing the injury incidence in youth team-sport players. Part 1: an analysis by classical training components
Source: Ann Med. 2024 Oct 1;56(1):2408457. doi: 10.1080/07853890.2024.2408457 (PMC11445890; doi:10.1080/07853890.2024.2408457)
Supplement: Supplemental Material [file IANN_A_2408457_SM0607.zip › suppl_data/Supplementary file 6.docx]

Supplementary file 6. Description of the 11 criteria designed to assess methodological quality of the studies included in the network meta-analysis with the PEDro scale.

| 1. Eligibility criteria were specified. |
| --- |
| 1. Subjects were randomly allocated to groups (in a crossover study, subjects were randomly allocated an order in which treatments were received). |
| 1. Allocation was concealed. |
| 1. The groups were similar at baseline regarding the most important prognostic indicators. |
| 1. There was blinding of all subjects. |
| 1. There was blinding of all therapists who administered the therapy. |
| 1. There was blinding of all assessors who measured at least one key outcome. |
| 1. Measures of at least one key outcome were obtained from more than 85% of the subjects initially allocated to groups. |
| 1. All subjects for whom outcome measures were available received the treatment or control condition as allocated or, where this was not the case, data for at least one key outcome was analysed by “intention to treat”. |
| 1. The results of between-group statistical comparisons are reported for at least one key outcome. |
| 1. The study provides both point measures and measures of variability for at least one key outcome. |
