# Supplementary material for: A systematic review and network meta-analysis on the effectiveness of exercise-based interventions for reducing the injury incidence in youth team-sport players. Part 1: an analysis by classical training components
Source: Ann Med. 2024 Oct 1;56(1):2408457. doi: 10.1080/07853890.2024.2408457 (PMC11445890; doi:10.1080/07853890.2024.2408457)
Supplement: Supplemental Material [file IANN_A_2408457_SM0607.zip › suppl_data/Supplementary file 7.docx]

| **Supplementary file 7.** Description of the 13 criteria designed to assess risk of bias of the studies included in the network meta-analysis with the Cochrane Back and Neck Group scale. | |
| --- | --- |
| **Bias domain** | **Source of bias** |
| Selection | (1) Was the method of randomization adequate? |
| Selection | (2) Was the treatment allocation concealed? |
| Performance | (3) Was the patient blinded to the intervention? |
| Performance | (4) Was the care provider blinded to the intervention? |
| Detection | (5) Was the outcome assessor blinded to the intervention? |
| Attrition | (6) Was the drop-out rate described and acceptable? |
| Attrition | (7) Were all randomized participants analyzed in the group to which they were allocated? |
| Reporting | (8) Are reports of the study free of suggestion of selective outcome reporting? |
| Selection | (9) Were the groups similar at baseline regarding the most important prognostic indicators? |
| Performance | (10) Were cointerventions avoided or similar? |
| Performance | (11) Was the compliance acceptable in all groups? |
| Detection | (12) Was the timing of the outcome assessment similar in all groups? |
| Other | (13) Are other sources of potential bias unlikely? |
