# Supplementary material for: A systematic review and network meta-analysis on the effectiveness of exercise-based interventions for reducing the injury incidence in youth team-sport players. Part 1: an analysis by classical training components
Source: Ann Med. 2024 Oct 1;56(1):2408457. doi: 10.1080/07853890.2024.2408457 (PMC11445890; doi:10.1080/07853890.2024.2408457)
Supplement: Supplemental Material [file IANN_A_2408457_SM0607.zip › suppl_data/Supplementary file 8.docx]

| **Supplementary file 8.** Component breakdown of the injury prevention programs. | | | | | | |
| --- | --- | --- | --- | --- | --- | --- |
| **Reference** | **Strength** | **Plyometrics** | **Stability** | **Speed/agility** | **Coordination / warm-up drills** | **Flexibility** |
| Achenbach et al. (2017) | Yes.  Nordic hamstring | Yes.  Multidirectional single-leg jumps, ice-skater jump, jump run | Yes.  SL stabilisation, plank, side plank | No. | No. | No. |
| Åkerlund et al. (2020) | Yes.  SL/DL squat, pelvic lift, forward lunge | Yes.  SL hop forward/backward, side-to-side hop (SL landing), DL jump with ball header | Yes.  Prone/side plank | No. | No. | No. |
| Al Attar et al. (2023) | Yes.  Press-ups, spiderman | Yes.  Skating jumps, SL jumps | Yes.  “Alertness” running game, Skating jumps, SL stance, press-ups, SL jumps, spiderman | No. | No. | No. |
| Azuma & Someya (2020) | No. | No. | No. | No. | No. | Yes.  Practical stretching exercises, without the use of any equipment, for the tight body parts |
| Barboza et al. (2019)^1^ | Yes.  SL/DL squats, walking lunges, etc. | Yes.  SL/DL forward, vertical, and lateral jumps, skating jumps, etc. | Yes.  Plank, skater figure, cross-country skiing, etc. | Yes.  Dribbling, sprint, etc. | Yes.  Skipping, tripling, low knee lift, high knee lift, heel flick, simulation with hockey stick, etc. | Yes.  Sun salutation, inchworm exercise, etc. |
| Emery et al. (2010) | Yes.  Nordic hamstring, walking lunges, calf raises, abdominal strength exercise | Yes.  SL jumps | Yes.  SL balance exercises and home exercise program using wobble boards | No. | Yes.  Combination of light forward and backward running, and/or side-side shuttle, and/or knee-high skipping, and/or dribbling | Yes.  Dynamic stretch components: calves, leg swings back/forth, side skip with arm throws to the side |
| Emery et al. (2007) | No. | No. | Yes.  Sport-specific balance training and home exercise program using wobble boards | No. | No. | Yes.  Static stretch, and dynamic stretch components |
| Hislop et al. (2017)^1^ | Yes.  Lunges, Nordic hamstring, etc. | Yes.  SL/DL jumps, etc. | Yes.  SL balance, side bridge, static side press up with perturbation, bear crawl with resisted static neck contractions, etc. | Yes.  Side shuffle, diagonal side shuffle, planned plant and cut, etc. | No. | No. |
| Imai et al. (2018) | No. | No. | Yes.  Bird dog (hand-knee), elbow-toe with raised arm and leg, and back bridge with one leg raised | No. | No. | No. |
| Junge et al. (2002) | Yes.  NA | Yes.  NA | Yes.  NA | Yes.  NA | Yes.  NA | Yes.  NA |
| Longo et al. (2012) | Yes.  Nordic hamstring, squats | Yes.  Jumping: vertical, lateral or box jumps | Yes.  Plank, side plank, SL balance | Yes.  Quick run, running over pitch, bounding run, running, and cutting | Yes.  Running: straight ahead, hip out, hip in, circling, running, and jumping | No. |
| Olsen et al. (2005) | Yes.  Nordic hamstring, DL squat, SL/DL squat on unstable surface | Yes.  Jump shot landing, bounding, forward jumps | Yes.  DL ball pass unstable surface, SL squat unstable surface, DL squat unstable surface, ball bounce with eyes closed unstable surface, perturbations on unstable surface | Yes.  Speed runs, planting  and cutting | Yes.  Jogging, backward running with sidesteps, forward running with knee and heel kicks, carioca, parade, forward running with trunk rotation, forward running with intermittent stops | No. |
| Owoeye et al. (2014) | Yes.  Nordic hamstring, squats | Yes.  Jumping: vertical, lateral or box jumps | Yes.  Plank, side plank, SL balance | Yes.  Quick run, running over pitch, bounding run, running, and cutting | Yes.  Running: straight ahead, hip out, hip in, circling, running, and jumping | No. |
| Rössler et al. (2018) | Yes.  Press-ups, spiderman | Yes.  Skating jumps, SL jumps | Yes.  “Alertness” running game, Skating jumps, SL stance, press-ups, SL jumps, spiderman | No. | No. | No. |
| Soligard et al. (2008) | Yes.  Nordic hamstring, squats | Yes.  Jumping: vertical, lateral or box jumps | Yes.  Plank, side plank, SL balance | Yes.  Quick run, running over pitch, bounding run, running, and cutting | Yes.  Running: straight ahead, hip out, hip in, circling, running, and jumping | No. |
| Steffen et al. (2008) | Yes.  Nordic hamstring | Yes.  SL hop forward/backward,  side-to-side hop, zigzag shuffle, bounding | Yes.  Cross-country  skiing, SL stance chest pass, SL stance forward bend, SL stance figure-of-8, prone/side plank | Yes.  Zigzag shuffle | No. | No. |
| Verhagen et al. (2023)^1^ | Yes.  Squats, shoulder muscle training exercises with elastic bands, etc. | Yes.  Squat jumps back/forward/sideways, drop jumps, etc. | Yes.  SL balance, SL balance with ball pass, prone/side plank, superman, etc. | Yes.  Relay race, acceleration/deceleration, etc. | Yes.  Jogging, skipping, heel flicks, bounce the ball together, etc. | Yes.  Inchworm exercise, reaching the net, etc. |
| Wedderkopp et al. (1999) | Yes.  Functional strength training for all major muscle groups | No. | Yes.  Exercises using ankle discs | No. | No. | No. |
| Zarei et al. (2018) | Yes.  Nordic hamstring, squats | Yes.  Jumping: vertical, lateral or box jumps | Yes.  Plank, side plank, SL balance | Yes.  Quick run, running over pitch, bounding run, running, and cutting | Yes.  Running: straight ahead, hip out, hip in, circling, running, and jumping | No. |
| Zarei et al. (2019) | Yes.  Press-ups, spiderman | Yes.  Skating jumps, SL jumps | Yes.  “Alertness” running game, Skating jumps, SL stance, press-ups, SL jumps, spiderman | No. | No. | No. |
| Zouita et al. (2016) | Yes.  Multiple-joint  exercises, such as the squat and the bench press | No. | No. | No. | No. | No. |
| ^1^ These programs include different exercises for each age group or individual. For a matter of space, only the most relevant exercises are mentioned in each component.  SL: single leg; DL: double leg; NA: Description of exercises not available. | | | | | | |
