# Supplementary material for: A systematic review and network meta-analysis on the effectiveness of exercise-based interventions for reducing the injury incidence in youth team-sport players. Part 1: an analysis by classical training components
Source: Ann Med. 2024 Oct 1;56(1):2408457. doi: 10.1080/07853890.2024.2408457 (PMC11445890; doi:10.1080/07853890.2024.2408457)
Supplement: Supplemental Material [file IANN_A_2408457_SM0607.zip › suppl_data/Supplementary file 9.docx]

| **Supplementary file 9.** Analysis of the selected studies’ reporting quality-CONSORT (*n* = 21). | | | | | | | | | | | | | | | | | | | | | | | | | | | | | | | | | | | | | | |
| --- | --- | --- | --- | --- | --- | --- | --- | --- | --- | --- | --- | --- | --- | --- | --- | --- | --- | --- | --- | --- | --- | --- | --- | --- | --- | --- | --- | --- | --- | --- | --- | --- | --- | --- | --- | --- | --- | --- |
| **Reference** | **1a** | **1b** | **2a** | **2b** | **3a** | **3b** | **4a** | **4b** | **5** | **6a** | **6b** | **7a** | **7b** | **8a** | **8b** | **9** | **10** | **11a** | **11b** | **12a** | **12b** | **13a** | **13b** | **14a** | **14b** | **15** | **16** | **17a** | **17b** | **18** | **19** | **20** | **21** | **22** | **23** | **24** | **25** | **Score** |
| Achenbach et al. (2017) | 0 | 1 | 1 | 1 | 1 | 0 | 0 | 0 | 0 | 1 | 0 | 0 | 0 | 0 | 1 | 0 | 0 | 0 | - | 1 | 1 | 0 | 0 | 0 | 0 | 1 | 1 | 1 | - | 0 | 0 | 1 | 0 | 1 | 0 | 0 | 1 | 14 |
| Åkerlund et al. (2020) | 1 | 1 | 1 | 1 | 1 | 0 | 1 | 1 | 1 | 1 | 0 | 1 | 1 | 1 | 1 | 0 | 1 | 1 | - | 1 | 1 | 1 | 1 | 0 | 0 | 1 | 1 | 1 | - | 1 | 1 | 1 | 1 | 1 | 0 | 1 | 1 | 29 |
| Al Attar et al. (2023) | 1 | 1 | 1 | 1 | 1 | 1 | 1 | 1 | 1 | 1 | 0 | 1 | 0 | 1 | 1 | 1 | 0 | 1 | - | 1 | 1 | 1 | 1 | 0 | 0 | 0 | 1 | 1 | - | 1 | 0 | 1 | 1 | 1 | 1 | 1 | 1 | 28 |
| Azuma & Someya (2020) | 0 | 1 | 1 | 1 | 1 | 0 | 1 | 1 | 1 | 0 | 0 | 0 | 1 | 0 | 0 | 0 | 1 | 0 | - | 1 | 1 | 1 | 1 | 1 | 0 | 1 | 1 | 1 | - | 1 | 1 | 1 | 0 | 1 | 1 | 0 | 0 | 22 |
| Barboza et al. (2019) | 1 | 1 | 1 | 1 | 0 | 1 | 0 | 0 | 1 | 1 | 0 | 1 | 0 | 0 | 0 | 0 | 1 | 0 | - | 1 | 1 | 1 | 1 | 0 | 0 | 1 | 1 | 1 | - | 0 | 0 | 1 | 0 | 1 | 0 | 1 | 1 | 19 |
| Emery et al. (2010) | 1 | 1 | 1 | 1 | 1 | 1 | 1 | 1 | 0 | 1 | 0 | 1 | 0 | 0 | 1 | 1 | 0 | 1 | - | 1 | 1 | 1 | 1 | 1 | 0 | 1 | 1 | 1 | - | 0 | 0 | 1 | 0 | 1 | 0 | 0 | 1 | 24 |
| Emery et al. (2007) | 1 | 1 | 1 | 1 | 1 | 1 | 1 | 1 | 0 | 1 | 0 | 1 | 0 | 1 | 1 | 1 | 0 | 1 | - | 1 | 1 | 1 | 1 | 0 | 0 | 1 | 1 | 1 | - | 0 | 0 | 1 | 0 | 1 | 0 | 0 | 1 | 24 |
| Hislop et al. (2017) | 1 | 1 | 1 | 1 | 1 | 1 | 0 | 0 | 1 | 1 | 0 | 1 | 0 | 0 | 1 | 1 | 1 | 1 | - | 1 | 1 | 1 | 1 | 1 | 0 | 1 | 1 | 1 | - | 0 | 0 | 1 | 0 | 1 | 1 | 0 | 1 | 25 |
| Imai et al. (2018) | 0 | 0 | 1 | 1 | 0 | 0 | 0 | 0 | 1 | 1 | 0 | 0 | 0 | 0 | 0 | 0 | 0 | 0 | - | 1 | 1 | 0 | 0 | 0 | 0 | 0 | 0 | 1 | - | 0 | 0 | 1 | 1 | 1 | 0 | 0 | 1 | 11 |
| Junge et al. (2002) | 1 | 0 | 1 | 1 | 1 | 1 | 1 | 1 | 1 | 1 | 0 | 0 | 0 | 0 | 0 | 0 | 0 | 0 | - | 1 | 1 | 1 | 1 | 0 | 0 | 0 | 1 | 0 | - | 0 | 0 | 0 | 0 | 1 | 0 | 1 | 1 | 17 |
| Longo et al. (2012) | 1 | 1 | 1 | 1 | 1 | 1 | 1 | 0 | 1 | 1 | 0 | 1 | 0 | 1 | 1 | 1 | 1 | 1 | - | 1 | 1 | 1 | 1 | 1 | 0 | 1 | 1 | 1 | - | 0 | 0 | 1 | 1 | 1 | 0 | 1 | 1 | 28 |
| Olsen et al. (2005) | 1 | 1 | 1 | 1 | 1 | 1 | 1 | 0 | 1 | 1 | 0 | 1 | 0 | 0 | 1 | 1 | 1 | 1 | - | 1 | 1 | 1 | 1 | 1 | 0 | 1 | 1 | 1 | - | 1 | 0 | 0 | 1 | 1 | 0 | 1 | 1 | 27 |
| Owoeye et al. (2014) | 1 | 0 | 1 | 1 | 1 | 1 | 0 | 1 | 1 | 1 | 0 | 0 | 0 | 0 | 1 | 1 | 0 | 0 | - | 1 | 1 | 1 | 1 | 1 | 0 | 1 | 1 | 1 | - | 0 | 0 | 1 | 0 | 1 | 0 | 1 | 0 | 21 |
| Rössler et al. (2018) | 1 | 0 | 1 | 1 | 1 | 1 | 1 | 1 | 1 | 1 | 0 | 1 | 0 | 1 | 1 | 1 | 1 | 1 | - | 1 | 1 | 1 | 1 | 1 | 0 | 1 | 1 | 1 | - | 0 | 0 | 1 | 1 | 1 | 1 | 1 | 1 | 29 |
| Soligard et al. (2008) | 1 | 1 | 1 | 1 | 1 | 1 | 1 | 0 | 1 | 1 | 0 | 1 | 0 | 0 | 1 | 1 | 1 | 1 | - | 1 | 1 | 1 | 1 | 1 | 0 | 0 | 1 | 1 | - | 1 | 0 | 1 | 1 | 1 | 1 | 1 | 1 | 28 |
| Steffen et al. (2008) | 1 | 0 | 1 | 1 | 1 | 1 | 0 | 0 | 1 | 1 | 0 | 1 | 0 | 0 | 1 | 1 | 1 | 1 | - | 1 | 1 | 1 | 1 | 0 | 0 | 0 | 1 | 1 | - | 0 | 0 | 1 | 0 | 1 | 0 | 1 | 1 | 22 |
| Verhagen et al. (2023) | 1 | 1 | 1 | 1 | 1 | 1 | 1 | 1 | 1 | 1 | 0 | 1 | 0 | 1 | 1 | 1 | 0 | 1 | - | 1 | 1 | 1 | 1 | 0 | 0 | 1 | 1 | 1 | - | 1 | 0 | 1 | 0 | 1 | 0 | 1 | 1 | 27 |
| Wedderkopp et al. (1999) | 1 | 0 | 1 | 1 | 0 | 0 | 0 | 0 | 0 | 1 | 0 | 0 | 0 | 0 | 1 | 0 | 0 | 0 | - | 1 | 1 | 0 | 0 | 0 | 0 | 0 | 1 | 0 | - | 0 | 0 | 0 | 0 | 1 | 0 | 0 | 1 | 10 |
| Zarei et al. (2018) | 1 | 1 | 1 | 1 | 1 | 1 | 1 | 1 | 1 | 1 | 0 | 0 | 0 | 0 | 0 | 0 | 0 | 0 | - | 1 | 1 | 1 | 1 | 0 | 0 | 1 | 1 | 1 | - | 0 | 0 | 1 | 1 | 1 | 0 | 1 | 1 | 22 |
| Zarei et al. (2019) | 1 | 1 | 1 | 1 | 1 | 1 | 1 | 1 | 1 | 1 | 0 | 1 | 0 | 1 | 0 | 0 | 1 | 1 | - | 1 | 1 | 1 | 1 | 0 | 0 | 1 | 1 | 1 | - | 0 | 1 | 1 | 1 | 1 | 1 | 1 | 1 | 28 |
| Zouita et al. (2016) | 0 | 1 | 1 | 1 | 0 | 0 | 1 | 0 | 0 | 1 | 0 | 0 | 0 | 0 | 1 | 0 | 0 | 0 | - | 1 | 1 | 0 | 0 | 0 | 0 | 1 | 0 | 0 | - | 0 | 0 | 0 | 0 | 1 | 0 | 1 | 0 | 11 |
